# Comparative Susceptibility to PM2.5-Induced Pulmonary Pathology: A Lifespan-Based Perspective on Animal Models

**DOI:** 10.3390/biology15212042

**Published:** 2026-11-09

**Authors:** Chutikarn Jutin, Wei-Hsiang Huang, Korakot Nganvongpanit, Jirapat Arunorat

**Affiliations:** 1 Faculty of Veterinary Medicine, Chiang Mai University, Chiang Mai 50100, Thailand; chutikarn_jutin@cmu.ac.th (C.J.); korakot.n@cmu.ac.th (K.N.); 2 Graduate Institute of Molecular and Comparative Pathobiology, School of Veterinary Medicine, College of Bioresources and Agriculture, National Taiwan University, Taipei City 10617, Taiwan; whhuang@ntu.edu.tw

**Keywords:** PM2.5, comparative pathology, animal model, sentinel species, One Health, pulmonary pathology

## Abstract

Fine particulate matter (PM2.5) is a leading environmental cause of respiratory disease, yet most evidence for its pulmonary toxicity comes from short-lifespan laboratory rodents exposed at high concentrations for a small fraction of their lives. The chemically heterogeneous components of PM2.5 converge on shared innate and adaptive inflammatory pathways across species. Whether these findings describe the disease that develops in long-lived animals breathing ambient air across decades remains unresolved. This review contrasts the phenotypic, cellular, and temporal manifestations of PM2.5-induced lung disease between short- and long-lifespan animals, across anatomical and physiological variables, time at risk, and immune response kinetics. Short-lifespan models express an acute, frequently reversible inflammatory injury, whereas long-lifespan species express a chronic phenotype of cumulative particle bioaccumulation, airway remodeling, and spontaneously arising neoplasia. These poles are not mutually exclusive: sufficient dose or observation time can drive short-lived models into the chronic phenotype, and lifespan remains confounded with exposure design, so it cannot yet be separated from exposure intensity and duration. The two groups are therefore best viewed as complementary, rather than competing, sources of evidence. Within a One Health framework, future priorities include long-term veterinary sentinel cohorts in high-exposure regions, cross-species dosimetric scaling, and direct in-tissue quantification of retained particle burden to link dose to lesion.

## Simple Summary

Fine particulate matter comprises airborne particles that are equal to or smaller than 2.5 micrometers, small enough to reach the lower airways and cause respiratory disease. Most mechanistic evidence of these particles derives from laboratory rodents, principally mice and rats. Humans, dogs, cats, and other animals sharing their environment inhale ambient air for years to decades, and it remains unclear how well findings from short-lifespan models describe disease that develops over such intervals. We compared the lung damage reported in short-lifespan animals with that reported in long-lifespan animals. Short-lifespan animals mostly show sudden inflammation that usually heals. In longer-lived animals, particles slowly accumulate in the lung tissue over exposures whose dose and duration are unknown, and observational studies report an association with permanent damage, including scarring and, in dogs, lung cancer. Because the two groups were studied in very different ways, these differences may reflect how much and how long the animals were exposed, as well as how long they live. We conclude that the two kinds of animal answer different questions and are most useful when used together.

## 1. Introduction

Air pollution is one of the major risk factors contributing to diseases, such as respiratory diseases, which are among the leading causes of human mortality. Air pollution is the contamination of the indoor or outdoor environment by any chemical, physical, or biological agent that modifies the natural characteristics of the atmosphere [1]. According to the World Health Organization, around 7 million deaths [1], mainly from noncommunicable diseases, are attributable to the joint effects of ambient and household air pollution. Several significant pollutants can cause harmful effects on health, including gaseous pollutants like nitrogen dioxide and sulfur dioxide, and particulate matter of various sizes that can be transported into the respiratory system of living organisms, causing a cellular response and leading to accumulation in the body. Nitrogen dioxide and sulfur dioxide can exert effects directly, such as airway irritation leading to bronchoconstriction and epithelial oxidative stress; moreover, these gases can undergo atmospheric reactions to form secondary particles, which in turn damage the respiratory system [2].

Particulate matter is any substance, except pure water, that exists as a liquid or solid in the atmosphere under normal conditions and is of microscopic or submicroscopic size but larger than molecular dimensions [2]. The aerodynamic diameter is used as the summary indicator of particle size. Particulate matter with an aerodynamic diameter of 2.5 µm or less, or fine particulate matter (PM2.5), has been the focus due to its inhalable properties and diverse toxicities. The resulting health effects have been reported to depend on the chemical composition and physical characteristics of PM2.5, which in turn depend on its sources [1,2,3,4]. Sources of PM2.5 emissions are numerous and can be divided into primary emissions, which are particles released directly into the atmosphere, and secondary emissions, which form when emitted gases either condense directly into particles or undergo chemical transformation into species that subsequently condense as particles [2]. Anthropogenic emissions are the focus, as they can be regulated through action plans developed by relevant organizations. The greatest contributors are the combustion of fossil fuels and biomass for daily use or industrial production, which mainly release carbonaceous particles into the atmosphere [3,4]. Exhaust and non-exhaust activities from combustion release varying proportions of carbonaceous particles [4]. Moreover, the types of fuel also contribute to different chemical compositions of PM2.5, and a correlation between the source attribution of PM2.5 and health burden has been reported [5]. Industrial activities are a major contributor to the presence of metals and engineered nanomaterials (ENMs) in the environment [4]. Some examples of the major natural sources include volcanic eruptions and wildfires [4]. Secondary particles mostly form through chemical reactions of gaseous pollutants emitted from primary sources, such as the transformation of nitrogen oxides and sulfur oxides in the atmosphere [1,4].

PM2.5 is a complex mixture of organic, inorganic, and biological components. Its toxicity stems not only from particle concentration but also from chemical composition. Strong evidence has established causal relationships between both chronic and acute PM2.5 exposure and all-cause mortality [6]. For example, chronic exposures to particles are related to pathologies such as asthma [7], Chronic obstructive pulmonary disease [8], atherosclerosis [9], arteriosclerosis, hypertension [10], dermal injury [11], and developmental toxicity affecting three major organ systems: the central nervous system, the male reproductive system, and the immune system [12,13,14].

Studies on PM2.5 toxicity have mostly been conducted in laboratory settings using short-lifespan animal models to study inflammatory mechanisms via acute response. Chronic responses have also been reported in recent years to emphasize the importance of air pollution control measures [15,16,17,18], but the chronic response in short-lifespan models may not represent the chronic lesions in long-lifespan species. Moreover, many individual components of PM2.5 have been studied and reported only in comparison with each other. As a result, studies on the overall toxicity of PM2.5 in long-lifespan animals remain very limited. In addition, data on the effects of chronic PM2.5 exposure under real-world ambient air pollution conditions, as opposed to controlled laboratory environments, remain similarly scarce. The objective of this review article is to contrast the phenotypic, cellular, and temporal manifestations of PM2.5-induced lung disease between short-lifespan and long-lifespan animals. In this review, short-lifespan animals are laboratory species with a median lifespan of approximately 3 years or less. Mice and rats represent this group because they are the most widely used species in toxicity studies. In contrast, long-lifespan animals are defined as those with a median lifespan of more than 10 years, a timescale closer to that of humans. This group includes companion animals such as dogs and cats, non-human primates, and zoo and wild animals. Studies in these species are largely naturalistic: exposure occurs in the real environment, so the dose, the particle composition and other conditions cannot be controlled, and most data come from samples collected from animals living in that environment. Species with intermediate lifespans (3–10 years), such as rabbits, guinea pigs and ferrets, were not assigned to either group because few PM2.5 studies exist in them. The exception is the guinea pig studies; they were grouped with the short-lifespan animals because their design was controlled in the same way as the other short-lifespan studies. Rabbits and ferrets are mentioned in Section 5 only to compare respiratory anatomy. Lifespan is also strongly confounded with study design in this review, because short-lifespan species are studied almost entirely in controlled experiments and long-lifespan species almost entirely through naturalistic observation. Lifespan is therefore used here to organize complementary bodies of evidence, not as a demonstrated causal determinant of phenotype, a point examined in Section 5.

This article is a narrative review and was not conducted according to a registered systematic-review protocol. Literature was identified by searching PubMed, Scopus, and Web of Science; authors searched from July to October 2026 for combinations of terms for fine particulate matter (for example, “PM2.5”, “fine particulate matter”, “air pollution”), lung pathology (for example, “lung injury”, “pulmonary fibrosis”, “anthracosis”, “lung cancer”), and animal species or models (for example, “mouse”, “rat”, “dog”, “cat”, “wildlife”, “sentinel species”), supplemented by the reference lists of key articles. Studies were included when they examined PM2.5, or closely related combustion-derived particles where no PM2.5 study was available, in an animal model and reported lung or respiratory outcomes, and when the species, exposure, and outcome were described well enough to be compared. Studies of PM10 alone, of non-respiratory outcomes only, and of human-only cohorts were used as background but not as comparative evidence. For Table 1 and Table 2, studies were chosen to be representative of each species, exposure route, or exposure design, giving priority to studies with the clearest exposure description, to studies that illustrate a distinct phenotype, and to the few studies that compare species directly [19]. For the short-lifespan models (Table 1), studies used PM2.5 that had been collected or characterized by the investigators. For the long-lifespan species (Table 2), PM2.5 was rarely measured directly, so studies of ambient or indoor air pollution, dust, or lung anthracosis as a marker of exposure were included as the closest available evidence, and each entry states the pollutant or exposure basis. This is a major limitation of the long-lifespan literature: the exposure is usually inferred from the setting and not quantified, so the lesions reported in these species generally cannot be attributed to PM2.5 specifically. The exceptions are a small number of studies that measured PM2.5 directly, namely a study of oxidative stress in the airways of dogs and studies of companion dogs and cats in Taiwan in which PM2.5 was measured daily [20,21,22].

Short-lifespan animals may not develop the outcomes observed in long-lifespan animals, such as neoplasia and particle accumulation, unless the dose is raised well above ambient levels, because these lesions require a prolonged dose–duration to develop. Long-lifespan animals are therefore better suited to addressing diseases that arise from cumulative exposure. The comparison focuses on anatomical and physiological variables, time-related risk factors, and variation in immune responses, all of which are important for translating findings into the One Health framework.

## 2. Pathophysiological Mechanisms of PM2.5 in the Respiratory System

### 2.1. Deposition

Particulate matter is removed from inspired air and deposited along the respiratory tract through five processes: impaction, interception, sedimentation, diffusion, and electrostatic attraction. Impaction occurs when large particles with high inertia cannot follow a deflected airstream and collide with the airway wall, and predominates at the front edge of the turbinates and the posterior wall of the nasopharynx, where airflow changes direction most abruptly [23]. Interception occurs when a particle becomes trapped in airway surfactant, mucus, glycocalyx, or cell membrane [23]. Sedimentation describes particles falling out of the airstream as airflow velocity decreases with the increasing total cross-sectional area of successive airway generations [23]. Particles that escape these mechanisms may still contact the airway wall by diffusion, which becomes the dominant mechanism for the smallest particles. Electrostatic attraction, determined by particle surface charge, increases migration toward the airway wall [23,24]. As a general rule, smaller particles deposit more distally, and fine and ultrafine fractions have been shown to translocate beyond the lung and deposit in multiple organs [13,25,26]. Deposition is not, however, determined by particle size alone. Particle composition and surface characteristics modify deposition behavior. Two other host factors, respiratory tract anatomy and breathing pattern, produce substantial differences in the distribution of delivered dose [24]. These two factors are the principal reason that equal ambient concentration does not correspond to equal delivered dose across species.

### 2.2. Clearance Mechanisms

Particles depositing on the conducting airway surface are trapped within a mucus gel layer composed of electrolytes and gel-forming mucin glycoproteins [27]. Mucin glycans, MUC5AC and MUC5B, bind microbial proteins and trap inhaled particles, thereby limiting their access to the epithelial surface [27]. Mucociliary clearance efficiency depends on the hydration state of the mucus layer, which is determined by an electrolyte balance comprising passive Na^+^ and water diffusion via CFTR and calcium-activated chloride channels. Epithelial ATP release fine-tunes this balance by stimulating Cl^−^ secretion and inhibiting Na^+^ absorption, and concurrently triggers surfactant release, indirectly supporting clearance and reducing surface tension [23]. Particles reaching the gas-exchange region beyond the ciliated airways are cleared principally by alveolar macrophages, which sit at the air–liquid interface and are the first responders to alveolar particle deposition [23,28,29]. Relative to interstitial and intravascular macrophages, alveolar macrophages are characterized by strong Fc-independent phagocytosis and by robust secretion of reactive oxygen species and cytokines upon activation [23]. Macrophage-mediated clearance is a slow, first-order process whose rate differs markedly between species. Clearance of the initial daily fraction of insoluble monodisperse particles transported out of the thorax in man, baboon, and dog was found to be slower than in rodents [30]. Particles activate macrophages through two receptor routes. Direct recognition occurs via scavenger receptors and pattern recognition receptors. The scavenger receptor family binds both inorganic ligands such as silica and titanium and organic ligands such as low-density lipoprotein; the classes SR-AI/II, MARCO, CD36, CD68 and LOX-1 are specifically implicated in the response to environmental particles [23]. Pattern recognition receptors, chiefly Toll-like receptors 2 and 4, bind pathogen- and damage-associated molecular patterns directly. Particles may also be recognized indirectly following opsonisation by immunoglobulins, complement, or collectins, including surfactant proteins A and D, engaging Fc receptors, complement receptors, or collectin receptors, respectively [23]. Following activation, macrophages phagocytose the particle and release reactive oxygen and reactive nitrogen intermediates together with pro-inflammatory cytokines and chemoattractants, and this cascade is recognized as the central mechanism of the classical dust-induced occupational pneumoconioses including silicosis, asbestosis and berylliosis, with the resulting pathology and mediator profile determined by particle characteristics [23].

Contemporary mechanistic work has resolved several of the specific pathways engaged by PM2.5. Phagocytosis of PM2.5 activates the NLRP3 inflammasome and drives release of IL-1β, with lysosomal rupture and potassium efflux mediating the release and mitochondrial Complex I-derived superoxide, rather than Complex III, driving the response; inflammasome priming and assembly were time- and dose-dependent and consistent across PM2.5 samples of variable chemical composition [31]. PM2.5 also drives macrophage phenotypic polarization. Su et al. reported that ambient PM2.5 promotes M1 (pro-inflammatory macrophage) polarization of alveolar macrophages in acute lung injury via activation of glutaminase 1-mediated glutaminolysis, with glutamate and succinate concentrations elevated in PM2.5-treated lungs and macrophages, implicating metabolic reprogramming as an upstream determinant of the inflammatory phenotype [32]. Critically, PM2.5 does not merely activate alveolar macrophages; it degrades the clearance function on which alveolar particle and pathogen removal depends. PM2.5 exposure hampered macrophage phagocytosis and pro-inflammatory cytokine production in response to pneumococcal infection, and in a PM2.5-exposed, pneumococcus-infected mouse model, PM2.5 subverted the pulmonary immune response, caused leukocyte infiltration, and suppressed CXCL10 and its receptor CXCR3 through inhibition of the PI3K/Akt and MAPK pathways. This created the net effect of enhancing pneumococcal infectivity and aggravating pulmonary pathogenesis [33]. Macrophage mobility declines as individual particle burden rises, and once alveolar macrophage-mediated clearance is overwhelmed, excessive lung burdens accumulate. The volume of rat alveolar macrophages is substantially greater than that of mice and hamsters, requiring a correspondingly greater number of engulfed particles to induce stasis, and it is noted that poorly soluble, low-toxicity particles produce tumorigenic responses only in rats and only once macrophage-mediated clearance is overwhelmed and chronic inflammation persists, raising unresolved questions about the relevance of these rodent findings to human carcinogenic risk [34].

### 2.3. Interstitialisation and Lymphatic Translocation

Alveolar macrophage clearance is neither complete nor exclusive. A proportion of deposited particulate escapes phagocytosis and crosses the alveolar epithelium into the pulmonary interstitium, and a further proportion is retained within macrophages that migrate interstitially rather than to the mucociliary escalator. Particle accumulation in lymph nodes was reported in many experimental studies, both demonstrating macrophage transport and direct particle passage operation [35,36]. Particles taken up into the interstitium may be re-entrained onto the airway epithelium [37]. This indicates that particles sequestered in this compartment may therefore be translocated via lymphatic drainage to the tracheobronchial lymph nodes, re-enter the clearance pathway after prolonged delay, or persist within interstitial and perivascular macrophage aggregates. The quantitative importance of this pathway differs profoundly between species. Nikula et al., exposing rats and cynomolgus monkeys concurrently for 24 months to diesel exhaust, coal dust, or both, found that rats retained a greater proportion of particulate material in the lumens of alveolar ducts and alveoli, whereas monkeys retained a greater proportion in the interstitium; monkeys retained a similar or greater total lung burden than rats, yet only the rats developed significant alveolar epithelial hyperplastic, inflammatory and septal fibrotic responses [19]. The rat acinus lacks respiratory bronchioles, possesses less interstitial connective tissue, and has pulmonary lymphatic vessels positioned differently from those of humans, with the consequence that different lung cells are predominantly in contact with the retained particles in the two species [38]. In the rat, the terminal bronchiole opens almost directly into the alveolar duct, so the inhaled particle stream decelerates and deposits in the proximal alveolar region, where the particle is met by a free alveolar macrophage lying on a thin alveolar septum. In macaques and humans, several generations of true respiratory bronchioles are interposed between the terminal bronchiole and the alveolar duct, and these airways are invested with a comparatively thick peribronchiolar interstitium containing bronchus-associated lymphoid tissue and the origins of the pulmonary lymphatic network. The primate particle is therefore delivered to a site that is immediately adjacent to a connective tissue compartment into which particle-laden cells can be transferred and from which they can drain, whereas the rat particle is delivered to a site from which the only efficient exit is back into the airway lumen. Comparative surveys of mammalian lung architecture confirm that this difference is a general one: the respiratory bronchiole and the interlobular septum, which are the anatomical substrates of the classical human dust lesion, are well developed in humans and in artiodactyls such as small pig breeds and goats but are poorly developed or absent in laboratory rodents, and this discrepancy is a recognized limitation on the transferability of rodent inhalation data [39]. Nikula et al. (2001) subsequently extended the comparison to human tissue and found that 57% of retained particulate material was interstitial in non-mining non-smokers, rising to 68% in non-smoking coal miners working under the 2 mg/m^3^ standard for a mean of 14 years, and to 91% in non-smoking miners who had worked under the former < 10 mg/m^3^ standard for a mean of 40 years [38]. This pathway is of central importance because it is the route by which retained carbonaceous particulate matter produces anthracosis: the accumulation of black carbon pigment within pulmonary interstitial macrophages, alveolar septa and draining lymph nodes [40].

Pulmonary anthracosis is the accumulation of carbonaceous particulate within lung tissue and its draining lymphoid tissue, and its pathogenesis follows directly from a single physical property of the material involved [40]. The sequence begins with deposition of the carbon core of combustion-derived air particles in the terminal bronchiolar and alveolar region. Because the phagocytosing cell cannot dispose of its load, the particle can only be relocated. A proportion of particle-laden macrophages is carried to the mucociliary escalator and removed, but in species in which macrophage-mediated clearance is comparatively weak, a substantial fraction instead migrates across the alveolar epithelium into the peribronchiolar and septal interstitium and drains along lymphatic routes to bronchus-associated lymphoid tissue and the tracheobronchial lymph nodes, where pigment accumulates in sinusoidal macrophages, predominantly in the medullary region [19,30,38,41]. Because the carbon is retained rather than eliminated, and because each successive exposure adds to a burden from which nothing is being subtracted, the deposit accumulates monotonically over the life of the animal [42]. Where local clearance is overwhelmed, unphagocytosed particles and the contents of lysed macrophages are released into the airway wall, initiating an inflammatory–oxidative–fibrotic cascade in which TGF-β1 signaling drives myofibroblast differentiation and matrix deposition; this converts simple pigment accumulation into anthracofibrosis [43]. Grossly, anthracosis appears as black to grey-black stippling, streaking or confluent discoloration of the pleural surface and the cut surface of the lung, typically most prominent along the interlobular septa, around the bronchovascular bundles and beneath the pleura—a distribution that traces the lymphatic drainage pathway rather than the airway tree [40]. The draining lymph nodes are correspondingly affected, with black pigmented areas prominent on their cut surfaces [41]. Histologically, the pigment appears as fine, granular, black, non-birefringent material within the cytoplasm of macrophages and lying free within the interstitium. The characteristic distribution is centred on the respiratory bronchiole and the surrounding interstitium in species that possess this structure, extending thence to the septa, pleura and hilar nodes; in the lymph node the pigment-laden sinusoidal macrophages are concentrated in the medulla [38,41]. Where fibrosis supervenes, collagen is laid down around the pigment to produce a pigmented fibrotic nodule, and in the airway wall the corresponding lesion is the pigmented, stenotic mucosa that defines bronchial anthracofibrosis at bronchoscopy, accompanied on computed tomography by bronchial narrowing or occlusion with wall thickening and calcified lymphadenopathy [43]. Simple pigment accumulation without fibrosis is generally not associated with symptoms or with measurable functional impairment, and it is routinely reported as an incidental finding; it is this benign behavior that makes it usable as an exposure marker [40]. However, the evidence clearly shows that anthracosis is not inert once the fibrotic threshold is crossed. Bronchial anthracofibrosis presents as an obstructive ventilatory defect with characteristically poor bronchodilator responsiveness—a consequence of the lesion involving large, cartilage-containing airways rather than the small airways affected in typical COPD—together with dyspnea and, in advanced cases, pulmonary hypertension; the fibrotic lesions are irreversible and respond poorly to current pharmacotherapy [43]. Between 27% and 60% of reported cases occur concurrently with pulmonary tuberculosis, and the anatomical overlap between the sites of stenosis and the predilection sites of tuberculosis has raised the possibility that the two conditions potentiate one another [43]. Anthracosis is also epidemiologically associated with tobacco exposure, air pollution and tuberculosis infection, and has been linked to mutations in the tumor suppressor genes P16 and P53 and to altered expression of CDH1, LUNX and RASSF1A, with affected individuals showing an increased frequency of pulmonary adenocarcinoma [44]. In veterinary studies, anthracosis in various species accordingly functions as a cumulative, integrative marker of lifetime particulate exposure rather than an index of recent dose [45,46,47,48]. Anthracosis serves as an air pollution exposure index in many studies [45,46,47,48,49]. Because the carbonaceous fraction of PM2.5 is retained as anthracotic pigment, histopathological assessment offers an accessible means of estimating retained particulate burden in tissue samples.

### 2.4. Water-Soluble and Organic-Soluble Fractions

PM2.5 can be classified into aqueous and organic extracts, based on solubility [3]. The water-soluble fraction is mainly composed of water-soluble organic matter, which can consist of oxygenated compounds, including keto-carboxylic acids, dicarboxylic acids, alcohols and aliphatic aldehydes, aromatic acids, saccharides, saccharide anhydrides, phenols, amines, amino acids, organic sulfates and nitrates, water-soluble trace metals (e.g., Ni, Cu, Mn, Fe) and ions (e.g., SO_4_^2−^, NO_3_^−^, K^+^, NH_4_^+^, PO_4_^3−^, Cl^−^, Na^+^) [50]. The water-insoluble fraction, in contrast, consists of kaolinite and calcium carbonate, Carbonaceous Matter, Polycyclic Aromatic Hydrocarbons (PAHs) and Inbound Trace Metals [51]. Water-soluble fractions have been shown to exhibit oxidative and immunomodulatory effects [50,52]. Water-soluble fractions readily dissolve in physiological fluids and enter the systemic circulation more efficiently than insoluble fractions, enabling direct interaction with extrapulmonary organs [53]. The water-insoluble fractions had a more pronounced effect on cell viability and membrane integrity than the water-soluble fractions, and both fractions induced similar levels of ROS in cells [52]. Exposure to water-soluble fractions triggers the release of pro-inflammatory cytokines such as IL-6, IL-8, and TNF−α across various cell models [50]. Water-insoluble fractions were more potent than water-soluble components in inducing Fe^2+^ release, ROS production, Malondialdehyde release, Glutathione depletion, and alterations in ferroptosis-related markers, which were detected through disruption of the Nrf2/Prdx6 pathway [51]. In chronic exposure studies, the water-soluble fraction, specifically sulfate content, acts as the primary driver of gut microbiome shifts, leading to the overrepresentation of sulfate-reducing bacteria within the Proteobacteria phylum [53].

### 2.5. Transition Metals

Transition metals play an important role in inducing oxidative stress conditions in cells. Metal constituents of PM2.5, such as Fe, V, Cu, Cr, Ni, Co, Pb, and Cd, increase the production of hydroxyl radicals through the Fenton reaction [25]. In addition, transition metals, including Ni, V, Co, and Cr, generate hydrogen peroxide and other highly reactive oxygen species (ROS) through the reduction of molecular oxygen in a multivalent state [25]. Exposure to nanoparticles coated with Ni and Fe induces changes in oxidative metabolism in the lung as reflected by increases in oxygen consumption, NOX activity, Thiobarbituric acid reactive substances (TBARS) assay levels and the GSH/GSSG ratio [54]. Transition metals cause not only oxidative stress, but also inflammatory response and DNA damage. IL-6 was significantly positively correlated with As and Pb, V and Cu from coal combustion-emitted PM2.5 [55]. TNF-*α* was significantly positively correlated with V, Cr, and Ni; both TNF-*α* and IL-6 play important roles in the development of inflammation [55]. IL-1β was found to be correlated with Co and Fe from bronchoalveolar lavage fluid following PM2.5 exposure [56]. A significant positive association between coal combustion and IL-1β level was observed, with a one-fold increase in this source associated with elevated IL-1β, suggesting that coal combustion was a major factor contributing to IL-1β secretion [56], consistent with the fact that coal combustion is a major source of heavy metal emissions. V and Cr in ambient air PM2.5 could play a major role in the induction of oxidative DNA damage by increasing 7-hydro-8-oxo-2′-deoxyguanosine (8-oxodG), which is believed to be a major product of hydroxyl radical attack on DNA [57]. Acute changes in lung function following exposure to transitional metal-rich PM2.5 show that Interquartile increases in Ca, Cd, Pb, Sr, Sn, V and Zn were associated with statistically significant increases in heart rate of 1–3 beats per minute, increases of 1–3 mmHg in blood pressure and lung function decreases of up to 4% in total lung capacity. This emphasizes the impact of transitional metals in PM2.5 and acute changes in cardiovascular and respiratory physiology [58]. Metal-bearing particles are also retained in human lung tissue: magnetite (Fe3O4) magnetic nanoparticles have recently been detected directly in the lung tissue of subway commuters in Hangzhou and Zhengzhou, and inhalation of such particles produced lung injury in mice [59].

### 2.6. Carbonaceous Particles

Carbonaceous particles (CPs) constitute a broad class of PM2.5 constituents that atmospheric scientists typically divide into two major categories based on their optical absorption properties: elemental carbon (EC) and organic carbon (OC). Elemental carbon comprises black carbon (BC), the strongly light-absorbing graphitic material formed during incomplete combustion, as well as engineered carbon nanomaterials such as carbon nanotubes, fullerenes, and graphene, which can enter ambient air through industrial and occupational sources. Organic carbon, by contrast, is a chemically diverse mixture that includes both primary organic compounds (emitted directly from combustion) and secondary organic compounds (formed through atmospheric oxidation of volatile precursors). Within this OC fraction, brown carbon (BrC) represents a particularly significant subgroup, arising from biomass burning, the degradation of organic matter, and secondary formation processes, and distinguished from BC by its weaker, wavelength-dependent light absorption concentrated in the UV and shorter visible range rather than BC’s broad, strong absorption across the visible-to-near-infrared spectrum. This EC/OC distinction matters mechanistically as well as optically, because BC and engineered carbon nanoparticles are chemically inert graphitic cores; their toxicity derives primarily from physical particle-membrane interactions and surface reactivity, whereas OC/BrC’s toxicity is compounded by the reactive organic chemistry of the compounds it comprises [4]. Mechanistically, isolating the carbon core from its adsorbed chemical cargo has suggested that CPs alone are sufficient to trigger acute pulmonary inflammation through a cell-type-specific pathway distinct from the classical macrophage-driven model. When C57BL/6 mice were exposed to carbon nanoparticles via intratracheal instillation, alveolar epithelial cells drove the earliest pro-inflammatory gene expression within the first 12 h, with macrophages remaining unstimulated during this initiating phase despite their role in subsequent particle clearance. This finding suggests that the epithelium, rather than resident immune cells, may act as the primary sensor of the bare carbon surface, although it rests on a single mouse instillation study and requires confirmation [60]. Uncoated carbon black nanoparticles (Printex^®^90) were compared against the same particles coated with benzo[a]pyrene, 9-nitroanthracene, or a mixed-PAH acetylene soot in a mouse model of established allergic airway inflammation. Notably, none of the carbon-black formulations, whether coated or uncoated, acutely worsened allergic inflammation at low dose; however, the uncoated carbon core alone reduced tracheal ciliary beat frequency and altered mucin (Muc5ac/Muc5b) expression, suggesting that the bare carbon particle may disrupt mucociliary clearance function independently of PAH chemistry, although this observation comes from a single study in allergic mice at low dose and requires replication [61]. At the chronic, occupational exposure end of the spectrum, ex vivo biosensor assays exposed primary human bronchial epithelial cells to serum from long-term carbon-black-exposed workers and found that chronic carbon black exposure activated cell-cycle-related pathways (via PLK1 and CDC20 gene expression) through a circulating, systemic inflammatory route, mechanistically distinct from the direct oxidative-stress-driven genotoxicity characteristic of PAH-laden diesel exhaust particle exposure in the same comparison [62]. Complementing this systemic route, serum from carbon-black-exposed workers activated vascular endothelial cells ex vivo, mediated by circulating proinflammatory factors, linking chronic carbonaceous particle exposure to cardiovascular as well as pulmonary risk [63]. Together, these studies indicate that the carbon particle core exerts epithelial-initiated acute inflammation, impaired mucociliary clearance, and systemic circulatory inflammation, driving chronic cell-cycle dysregulation.

### 2.7. Polycyclic Aromatic Hydrocarbons (PAHs)

Polycyclic aromatic hydrocarbons (PAHs) are a class of organic compounds composed of two or more fused aromatic rings, formed primarily as byproducts of incomplete combustion [1]. Within PM2.5, PAHs exist adsorbed onto the surface of the carbonaceous particle core rather than as free molecules, which allows them to be carried deep into the alveolar region alongside the particle itself. Representative PAH species identified in ambient PM2.5 include benzo[a]pyrene (BaP), benzo[e]pyrene, fluoranthene, benzo[b]fluoranthene, and indeno [1,2,3-cd]pyrene [64]. The primary sources of PAH-laden PM2.5 are combustion-related; PAH content in PM2.5 tends to track closely with a region’s dominant combustion sources rather than being a fixed proportion of particle mass [65,66]. PAHs exert their effects on respiratory cells primarily through activation of the aryl hydrocarbon receptor (AhR), a cytosolic receptor that, once bound, translocates to the nucleus and upregulates the cytochrome P450 enzymes CYP1A1 and CYP1B1 [67]. This receptor-mediated pathway serves as the branch point for several distinct downstream consequences. First, CYP1A1/CYP1B1-mediated metabolism of BaP produces a diol-epoxide intermediate capable of forming stable DNA adducts, directly linking PAH exposure to a genotoxic, pro-carcinogenic endpoint via an AhR/Src/ERK signaling axis [64]. Second, in primary human bronchial epithelial cells, PAH exposure induces both innate and adaptive immune signals, including IL-1A, IL-19, STAT6, and HLA-DMB/DRA, alongside oxidative stress response genes such as HMOX1 and NQO1, indicating that AhR activation simultaneously drives immune activation and antioxidant compensation [67]. Third, this immune/inflammatory response is not fixed but is modified by the underlying inflammatory state of the tissue. In a 3D organotypic bronchial model, cells exhibiting an asthmatic (IL-13-driven) phenotype showed heightened CYP1B1 induction upon PAH exposure compared to normal cells, suggesting that pre-existing airway inflammation sensitizes tissue to PAH-driven metabolic activation [68]. Fourth, PAHs disrupt epithelial barrier integrity through a mechanism distinct from receptor-driven immune signaling. The PAH-containing organic extract of PM2.5 collected during haze events in China caused pulmonary epithelial barrier dysfunction in BEAS-2B cells through depletion of tight-junction proteins from the zonula occludens, alongside increased DNA damage, proinflammatory cytokine release, and decreased α1-antitrypsin expression. Together, these effects have been proposed as a specific mechanism increasing COPD risk [69]. Finally, the relationship between PAH exposure and cell survival is not uniformly pro-injury. At low concentration levels, non-cytotoxic doses more representative of ambient exposure, the PAH fraction of PM2.5 was shown to protect human bronchial epithelial cells from mitochondria-driven apoptosis via the same AhR pathway, an effect not reproduced by endotoxin or carbon black alone [70], which is mechanistically significant because cells that fail to undergo apoptosis despite DNA damage are more likely to persist and progress toward malignant transformation, linking this seemingly protective effect back to the carcinogenic potential.

### 2.8. Microbial/Biological Components

Biological components can consist of suspended airborne particles released from living organisms, including bacteria, fungi, viruses, and their fragments [71,72]. These fragments include endotoxin or so-called lipopolysaccharide (LPS), which can trigger an allergic response in the respiratory airway [71]. These bioaerosols can be generated due to several natural and man-made activities, and their sources can emit different types and proportions of bioaerosols [72,73]. Meteorological factors and spatiotemporal factors are significantly related to changes in bacterial community structure [74]. The most abundant classified bacterial species in PM2.5 collected during the haze period in Beijing was *Geodermatophilus obscurus*, a bacterium commonly found in dry soil environments. This finding is consistent with, and may be partly attributed to, the lack of vegetation coverage and abundance of dry, exposed soil, and construction sites in the city of Beijing and its surrounding areas, especially during the winter seasons [73]. While the majority of the inhalable microorganisms were soil-associated and nonpathogenic to humans, pathogens were also found in the same PM2.5 samples, including *Streptococcus pneumoniae*, *Aspergillus fumigatus*, and human adenovirus C [73]. This report supports that the biological composition should also be a focus of disease-contribution investigation. Instillation of dairy barn coarse PM into mouse lungs results in an increase in inflammatory cells in bronchoalveolar lavage fluid (BALF) after exposure, an effect predominantly caused by heat-labile material, especially endotoxin [75]. The impact of LPS-induced pulmonary inflammation is associated with the quantity of LPS exposure [76]. When low levels of LPS are inhaled, this leads to Th2 cell induction, and IL-5 and IL-13 released by Th2 cells contribute to asthma development. In contrast, exposure to high levels of LPS induces a Th17 cell response, leading to increased production of IL-17, which can contribute to asthma, COPD, and lung cancer [76]. Air pollution impacts the respiratory microbiome through both direct physical interactions with microbes and indirect mechanisms such as oxidative stress, inflammation, and epithelial barrier disruption [76].

### 2.9. Convergent Innate Immune Activation

Regardless of which specific component initiates the response, whether it is the water-soluble fraction, transition metals, CPs, PAHs, or bioaerosols, the downstream signaling cascade converges on a shared set of pattern recognition receptor (PRR) pathways, indicating that the diverse composition of PM2.5 ultimately funnels into a common innate immune mechanism. PM2.5 was demonstrated to activate the NLRP3 inflammasome and caspase-1 activation in pulmonary tissue, a cytosolic sensing pathway that assembles in response to cellular stress signals such as ROS and lysosomal destabilization, both of which are generated by multiple PM2.5 components described above, and drives the maturation and release of the pro-inflammatory cytokines IL-1β and IL-18 [77]. In parallel, PM2.5 exacerbated allergic lung inflammation in mice through the TLR2/TLR4/MyD88 signaling axis, a membrane-bound receptor pathway that recognizes microbial-associated molecular patterns as well as damage-associated molecular patterns released from injured cells [78]. Reinforcing this convergence, whole PM2.5 can aggravate airway inflammation in asthmatic mice by activating NF-κB specifically through MyD88-dependent signaling, using TLR2, TLR4, and MyD88 knockout mice to confirm that cytokine and neutrophil responses were substantially attenuated when this axis was genetically disrupted, directly establishing causality rather than mere correlation [79]. Moreover, PM2.5 exacerbated oxidative stress and inflammation through simultaneous engagement of the Nrf2 antioxidant pathway and NF-κB in an allergic rhinitis mouse model, indicating that the same exposure event triggers both a pro-inflammatory response and a counter-regulatory antioxidant response in parallel [80]. The NLRP3/caspase-1 and TLR/MyD88/NF-κB pathways function as the convergence point through which the heterogeneous, component-specific mechanisms are ultimately translated into a unified innate inflammatory response.

### 2.10. Adaptive Immunity Effects

Beyond the innate immune activation described above, PM2.5 exposure also disrupts the balance of the adaptive immune system, compounding the innate inflammatory cascade. Intranasal instillation of PM2.5 in a rat model produces a measurable imbalance in the Th1/Th2 and Treg/Th17 cell populations alongside airway epithelial damage, with these adaptive immune shifts associated with aryl hydrocarbon receptor pathway activation, the same receptor implicated in PAH-driven epithelial injury [81]. Urban dust enhances Th17 polarization and IL-17A production in a dose-dependent manner in wild-type mice but fails to do so in aryl hydrocarbon receptor-deficient animals, which establishes the receptor as necessary to the effect rather than merely associated with it [82]. The opposing regulatory arm is suppressed at the same time. In children living in a high-pollution region, regulatory T cells showed impaired suppressive function and reduced chemotaxis together with significantly increased methylation at the FOXP3 locus, and the degree of regulatory T-cell impairment tracked asthma severity [83]. PM2.5 therefore does not simply shift the Th1/Th2 ratio; it amplifies a pro-inflammatory effector lineage through a defined receptor while epigenetically disabling the brake that would ordinarily restrain it. The T-cell imbalance is accompanied by altered immunoglobulin production, with PM2.5 exposure elevating circulating IgM, IgG, and IgE while suppressing IgA, changes that favor a hypersensitivity-prone immune profile rather than the mucosal-protective profile associated with IgA [84]. A heightened systemic and allergic antibody response with a weakened mucosal one, and the loss of IgA is mechanistically coherent with the epithelial and antimicrobial-peptide deficits described above, since secretory IgA performs exclusion at the same interface at which β-defensins, surfactant protein D and tight junction integrity are being lost [85]. The combination of distorted T-helper cell populations and elevated immunoglobulin production creates the immunological substrate for cytokine storms and allergic hypersensitivity reactions following repeated PM2.5 exposure, linking this adaptive dysregulation directly to asthma and allergic airway phenotypes.

### 2.11. From Inflammation to Structural Change

The innate and adaptive immune mechanisms described above are not confined to transient inflammation. When activated repeatedly or chronically, they drive a transition toward permanent structural remodeling of the airway. PM2.5 can induce airway remodeling through activation of the Wnt5a/β-catenin signaling pathway in a chronic obstructive pulmonary disease model, directly linking sustained PM2.5 exposure to the same structural pathway implicated in human COPD pathogenesis [86]. Extending this mechanism to a specific cell type, PM2.5 further induced both lung inflammation and fibrosis via airway smooth muscle cell expression of the Wnt5a/JNK pathway, with matched findings in human COPD bronchoalveolar lavage fluid samples, indicating that airway smooth muscle cells are an active mechanistic contributor to remodeling rather than a passive structural bystander [87].

**Figure 1:**
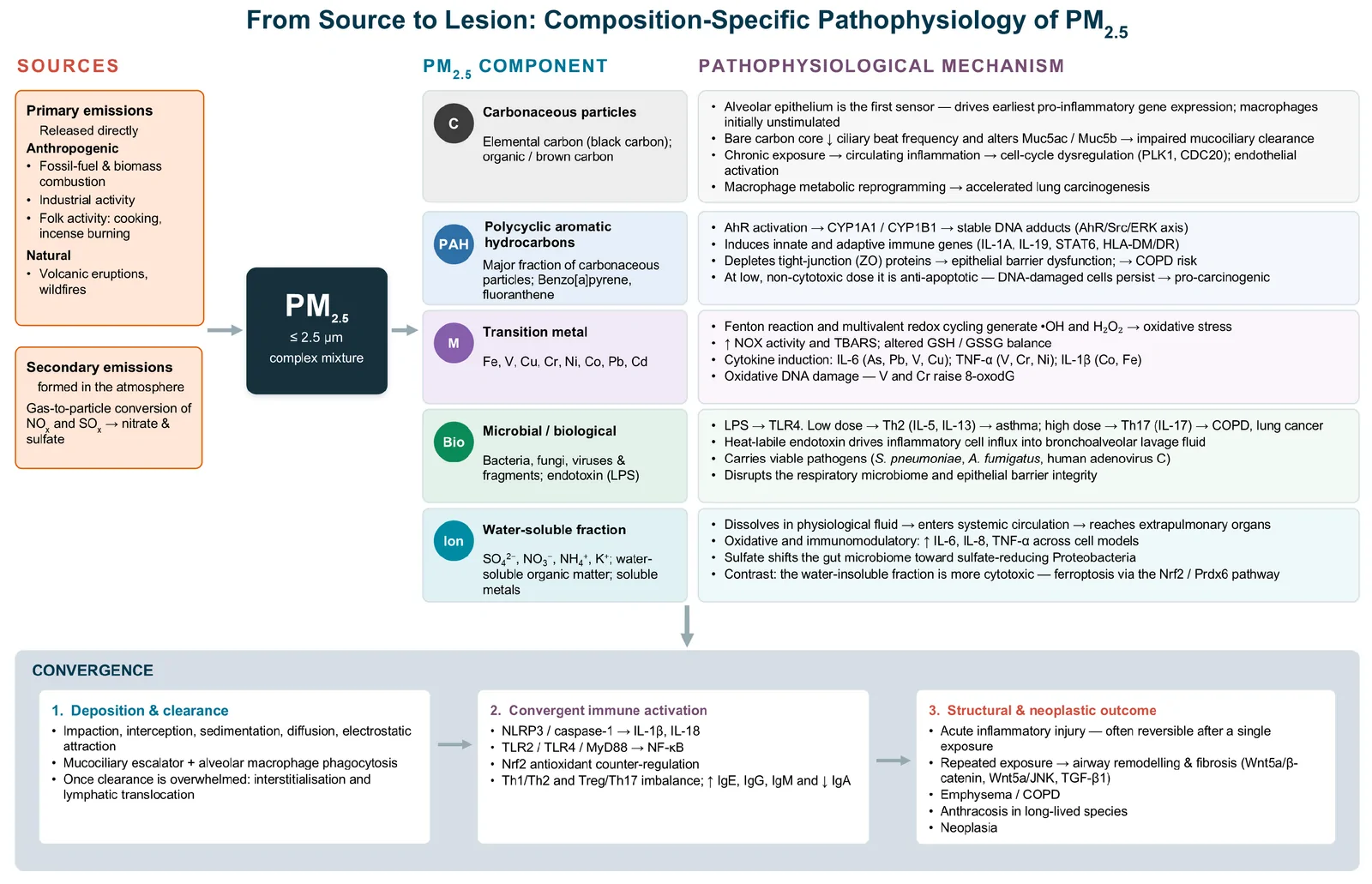
Composition-specific pathophysiology of PM2.5. Each component class acts through its own dominant mechanism, and all converge on a common innate and adaptive inflammatory program. Left, primary (anthropogenic and natural) and secondary sources of PM2.5. Centre and right, the five component classes considered in Section 2.4, Section 2.5, Section 2.6, Section 2.7 and Section 2.8 (carbonaceous particles, polycyclic aromatic hydrocarbons, transition metals, microbial/biological components, and the water-soluble fraction) and the dominant mechanism reported for each; several of these findings, notably those for the carbonaceous core (epithelial-initiated inflammation and reduced ciliary beat frequency), come from single studies (Section 2.6). Bottom, convergence of these routes on (1) deposition and clearance (Section 2.1, Section 2.2 and Section 2.3), (2) a shared innate and adaptive immune response (NLRP3/caspase-1 and TLR2/TLR4–MyD88–NF-κB signaling, Nrf2 counter-regulation, and Th1/Th2 and Treg/Th17 imbalance; Section 2.9 and Section 2.10), and (3) structural and neoplastic outcomes that repeated or prolonged exposure can produce (Section 2.11); anthracosis is reported mainly in long-lifespan species (Section 2.3 and Section 4.3), and neoplasia has been reported at high dose in rodents and as an observational association in dogs (Section 3.3 and Section 4.3). Arrows (→) indicate a causal or sequential step; ↑ and ↓ indicate increased and decreased levels or activity.

To conclude, the pathophysiology of PM2.5 is governed first by three physical determinants that precede any biological response: deposition, clearance and retention. PM2.5 disrupts each of these three physical steps rather than merely passing through them. Its size distribution shifts deposition distally, into a region served only by the slower macrophage-dependent pathway; it is reported to impair that pathway by reducing ciliary beat frequency and altering mucin expression (each shown in single studies) and by suppressing macrophage phagocytic capacity and mobility; and by doing so it converts a transient exposure into a retained interstitial burden that sustains chronic stimulation. Superimposed on this physical framework, the chemically heterogeneous constituents of PM2.5—the water-soluble and organic-soluble fractions, transition metals, the carbonaceous core, adsorbed polycyclic aromatic hydrocarbons, and biological components—initiate injury through distinct proximal routes, yet the resulting pathology can be resolved into six convergent mechanisms (Figure 1). First, oxidative stress arises as the most broadly shared response, generated by Fenton-type redox cycling of transition metals, by mitochondrial superoxide production, and by the reactive organic chemistry of the soluble fraction, with the Nrf2 axis engaged as counter-regulation. Second, innate inflammation follows, converging on NLRP3 inflammasome–caspase-1 activation and on TLR2/TLR4–MyD88–NF-κB signalling, which ultimately funnel into a common receptor-level response. Third, sustained innate activation propagates to adaptive-immune imbalance, expressed as distortion of the Th1/Th2 and Treg/Th17 axes and a shift in immunoglobulin profile. Fourth, barrier disruption and impaired clearance act as both an effect and an amplifier, as tight-junction depletion, ciliary dysfunction, and macrophage suppression increase epithelial permeability while prolonging particle and pathogen residence time. Fifth, repeated engagement of these pathways drives structural remodeling and fibrosis through Wnt5a/β-catenin and Wnt5a/JNK signaling, marking the transition from reversible inflammation to fixed architectural change. Sixth, genotoxic and carcinogenic mechanisms operate in parallel, through AhR/CYP1A1–CYP1B1-mediated formation of PAH–DNA adducts, hydroxyl radical–driven oxidative DNA damage, and apoptotic suppression that permits damaged cells to persist. These six mechanisms are not sequential alternatives but overlapping and mutually reinforcing processes.

## 3. Short-Lifespan Animals: Acute Dynamics and Accelerated Phenotypes

### 3.1. Short-Lifespan Representative Species

Short-lifespan animal models were selected based on their short median lifespan, typically less than three years, and their maintenance under controlled laboratory conditions [88,89]. Mice and rats are the two main species chosen for experimental studies on PM2.5-induced pulmonary pathology. These short-lifespan animal models can be customized to fit researchers’ specific mechanistic focus. Transgenic and knockout models allow researchers to study a chosen aspect of PM2.5 toxicity in depth. For example, mice with macrophage-specific NLRP3 deficiency are used to investigate the pathway of oxidative stress [31]. Short-lifespan animal models offer benefits such as shorter experimental timelines, lower cost, and the ability to control husbandry conditions. Controlled factors allow outcomes to be more directly attributable to the primary factor of interest, namely PM2.5 exposure. Collectively, this group is used to define acute inflammatory and molecular mechanisms, to isolate individual signaling pathways through genetic manipulation, and to establish dose–response relationships under controlled exposure; given sufficient dose or observation time, chronic and neoplastic endpoints can also be reproduced. Studies about how PM2.5 exposure can enhance the severity of other respiratory diseases have been conducted using mice with COPD or bacterial pneumonia as the experimental models [17]. Specific key findings on the mechanism can then be compared to determine whether they are correlated. To understand the acute response to PM2.5, in which proteins, cells, or chemical agents react to this external stimulus, mice and rats are the most commonly used species. Chronic remodeling studies also use these species, as they can be more easily manipulated. Fibrotic and neoplastic changes have been found in studies using mice [15,18,86,87]. Other short-lifespan mammals have also been used in PM2.5 toxicity research. Guinea pigs have been used in an allergic airway animal model involving PM2.5 exposure [90]. Guinea pigs have an intermediate lifespan and are included here because they were studied under the same controlled-exposure design as the rodents (see Section 1). Zebrafish were used to observe acute responses across multiple organ systems, including the cardiovascular, respiratory, hepatic, neurologic, and reproductive systems, and abnormal embryo development [91,92,93,94]. Non-mammalian animals were also considered as animal models. *Drosophila melanogaster* was chosen to study oxidative stress signaling [95]. These chosen animals, whose median survival is approximately two years, cannot be exposed to ambient concentrations for a biologically meaningful fraction of their lives and still yield measurable pathology within the timeframe of a fundable project; this constraint therefore drives species selection toward elevated dose, abbreviated exposure, and sacrifice at or shortly after the end of exposure. In retrospective studies of short-lifespan animals, terrestrial birds have been observed to analyze the impact of ambient air pollution on their life cycle at the population level [96]. Still, the comparability and interpretation of findings between short-lifespan animals and humans remain under discussion. It has been noted explicitly, in the context of acute lung injury modeling, that small animal models may be anatomically and physiologically inadequate proxies for larger species, and that failures in clinical translation have been attributed in part to these differences [34,88,89].

### 3.2. Acute Exposure Characteristics

Exposure protocols in short-lifespan models fall into two broad categories. Whole-body or nose-only inhalation using concentrated ambient particle systems (CAPS) most closely mimics real-world, route-of-entry exposure but leaves the delivered pulmonary dose difficult to quantify precisely. Intratracheal or intranasal instillation, by contrast, delivers a defined bolus mass of PM2.5 directly to the lower respiratory tract and is favored when dose–response relationships must be established. It offers precise dosimetry and has been used to establish some of the most mechanistically informative findings in the field [78]. The exposure protocols used in these studies are typically established based on the study objectives and practical convenience; for example, chronic exposure studies often use repeated low-dose exposure, whereas acute studies use short-term high-dose exposure. Acute, short-term exposure typically produces lesions that resolve after exposure ends [97], whereas chronic, repeated exposure results in clear tissue remodeling [15,18,86,87]. Because rodent lifespans are already short relative to humans, even subchronic protocols represent a substantial fraction of the animal’s total lifespan, effectively compressing years of cumulative human exposure into a period of weeks—an important caveat when extrapolating kinetics to human chronic disease [88,89]. In Drosophila and zebrafish, exposure is typically delivered via aerosolized or dietary concentrated ambient particles and waterborne PM2.5 suspensions, respectively, allowing near-complete lifespan or whole-embryonic-development coverage within the timeframe of a single assay [94,95]. The exposure durations of the representative studies in Table 1 indicate that most protocols occupy between 0.4% and 15% of the animal’s median lifespan, assuming median laboratory lifespans of approximately 730 days for mice and 1000 days for rats [98,99]. The percentages given in Table 1 are indicative only and would change in inverse proportion to the lifespan assumed. Table 1 also lists the concentration or dose reported by each study; because these are expressed in different units (µg/m^3^ for inhalation, µg or mg per animal for instillation), they cannot be compared directly, which is itself a limitation of the exposure data available for these models. Studies described as chronic in the rodent literature, for instance showing three months of continuous exposure, correspond to roughly one-eighth of a mouse’s lifespan [16]. Whether such a design models chronic human exposure, which may extend across seventy years, is not resolved by the descriptor alone.

**Table 1:** Representative studies of PM2.5-induced pulmonary and systemic effects in short-lifespan animal models, compared by species, exposure design, and disease phenotype.

Species/Model	Particle Type/Source	Route	Concentrations/Dose (as Reported)	Duration (≈% Median Lifespan)	Primary Phenotype/Outcome	Primary Research Focus
Mouse, male ICR [100]	Whole particle/analyzed ambient PM2.5	Whole-body inhalation	435.43, 342.83 and 382.18 µg/m^3^ for the 30-, 60- and 90-day exposures, respectively	30/60/90 d (≈4–12%)	Oxidative stress & genotoxic damage—DNA damage with impaired DNA repair Innate inflammation—progressive, not plateauing Structural remodeling—fibrosis; all endpoints rise with exposure duration	Temporal—duration-response
Mouse, C57BL/6 [75]	Whole particle/dairy-barn PM, bioaerosol-rich (endotoxin); fine vs. coarse	Intratracheal instillation	50 µg per mouse (single instillation; fine and coarse fractions)	Single dose; assessed 24 h & 72 h (<1%)	Innate inflammation—neutrophil infiltration peaking at 24 h, then resolved by 72 h	Mechanistic—outcome after acute exposure
Mouse, BALB/c WT & TLR2/TLR4/MyD88-KO [78]	Whole particle/urban ambient PM2.5 (± ovalbumin)	Intratracheal instillation	0.1 mg (100 µg) per mouse per instillation; 4 instillations	4 instillations at 2-week intervals (≈6 wk; ≈6%)	Adaptive immune imbalance/hypersensitivity—eosinophilic airway inflammation; ↑ IL-5, IL-13, IgE Innate inflammation—TLR2/TLR4/MyD88-dependent	Mechanistic—knockout lines isolate the initiating receptor pathway
Mouse [16]	Whole particle/ambient PM2.5 alone (no cigarette smoke)	Intratracheal instillation	0.59 mg per mouse (200 µL, ≈2.96 mg/mL)	Once weekly for 3 months (≈12%)	Structural remodeling—emphysema on micro-CT with declining respiratory function showing a full COPD phenotype	Mechanistic— establishes PM2.5 alone as sufficient to reproduce COPD
Mouse, PM2.5-induced COPD model [18]	Whole particle/biomass-burning PM2.5	Whole-body inhalation	6 h/day; PM2.5 concentration not reported (organoid experiments: 50 µg/mL)	6 months (≈25%)	Structural remodeling—reduced pulmonary compliance and emphysema via impaired AT2 → AT1 transdifferentiation	Mechanistic— identifies a progenitor-cell defect rather than inflammation alone
Rat, Wistar [81]	Whole particle/PM2.5 suspension	Intranasal instillation	0.3 mg/mL suspension; daily for 15 d	15 d exposure + 75 d observation (≈1.5%)	Adaptive immune imbalance—Th1/Th2 and Treg/Th17 shift with AhR activation Barrier disruption—airway epithelial damage and infiltration	Mechanistic & intervention—inflammatory effects and the effect of oral bacterial-lysate treatment
Rat, Sprague-Dawley [101]	Whole particle/ambient PM2.5	Intratracheal instillation (repeated)	1.8, 5.4, 16.2 mg/kg body weight	1 month (10 exposures) + 18-month follow-up	Structural remodeling—collagen and hydroxyproline continue rising throughout washout and fibrosis progresses after exposure ends	Temporal (post-exposure latency)—separates trigger from disease progression
Guinea pig, Cavia porcellus, OVA-sensitized [90]	Whole particle/concentrated ambient PM2.5	Whole-body inhalation	609 ± 12.73 µg/m^3^; 5 h/day	5 d, Acute, during sensitization/challenge	Adaptive immune imbalance/hypersensitivity—↑ IgE and IgG1 with worsened airway hyperresponsiveness; result shows PM2.5 acts as an immunological adjuvant	Susceptibility—effect of pre-existing allergic airway disease
Zebrafish, Danio rerio (embryo/larva) [94]	Whole particle/PM2.5 suspension	Waterborne exposure	25, 50, 100, 200 µg/mL, for cardiotoxicity and hepatotoxicity assays 25, 50, 100, 200, 400 µg/mL, for embryonic tests	Embryonic–larval window (days)	Multi-organ and developmental injury—↑ mortality, ↓ hatching rate; histopathology in heart, liver, gut and muscle	Mechanistic—developmental toxicity
*Drosophila melanogaster* [95]	Whole particle/ambient PM2.5	Whole-organism inhalation chamber	52–91 µg/m^3^; 6 h/day, 5 days/week	Continuous, ≈full 6–8-wk lifespan (≈100%)	Oxidative stress & innate inflammation—Jak/JNK/NF-κB activation Shortened lifespan—survival itself as the toxicological endpoint	Whole-organism survival—observe full-lifespan coverage to determine survival endpoint

↑, increased; ↓, decreased; →, leads to.

### 3.3. Short-Lifespan Model Disease Phenotype

The dominant phenotype in short-lifespan models is an acute-onset, dose-dependent inflammatory injury: neutrophilic and macrophagic infiltration of the airways and alveolar interstitium, alveolar epithelial damage, apoptosis, and a rapid proliferative repair response driven by type II pneumocytes [32,60,102]. Importantly, acute injury of this kind is frequently reversible [75,97]. Rodent models can and do produce the chronic and neoplastic phenotypes conventionally attributed to long-lived species, provided exposure is sufficiently intense or observation sufficiently prolonged. With repeated or prolonged instillation, this acute pattern progresses toward incipient interstitial fibrosis, marked by rising type I/III collagen deposition, α-smooth muscle actin expression, and TGF-β1 signaling, together with measurable declines in total lung capacity and compliance [87,101]. Lung neoplasms developed in a mouse model exposed to PM2.5 collected from ambient South Korean air [15]. Chronic exposure to carbon black ultrafine particles reprogrammed macrophage metabolism and accelerated lung cancer development [103], linking sustained particulate burden and macrophage dysfunction to carcinogenesis. These phenotypes are produced by experimental designs built specifically to compress prolonged exposure into a rodent lifespan. Such protocols deliver a calculated particle concentration through repeated or continuous exposure over weeks to months, and some extend observation well beyond the exposure period so that late-developing lesions can emerge [15]. Assessment then combines in vivo and terminal measures. At the end of the study, the lungs are examined histopathologically, with fibrosis quantified by collagen and hydroxyproline content, α-smooth muscle actin expression and TGF-β1 signaling [15,87,101], and neoplastic change recorded as the incidence of tumors and pre-neoplastic foci [15,103]. Cumulative genotoxic damage is measured in parallel through markers of DNA damage and impaired DNA repair [100]. Because most designs terminate at or shortly after the end of exposure, these histopathological endpoints represent a terminal snapshot rather than a longitudinal record, and standardized semi-quantitative lesion scoring is what allows burden to be compared across studies [15]. The composition of the particulate determines the character of this response. In animals with pre-existing COPD-like disease, PM2.5 exposure clearly exacerbates the baseline phenotype, demonstrating that short-lifespan rodents can model susceptibility [17]. A related susceptibility phenotype appears in allergic-asthma guinea pig models, where PM2.5 acts as an immunological adjuvant, amplifying IgE/IgG1 production and airway hyperresponsiveness upon allergen challenge [90]. The non-mammalian models contribute complementary, organism-level phenotypes: zebrafish embryos show increased mortality, inhibited hatching, and histopathological changes in various systems [91], while Drosophila exhibit measurably shortened lifespan itself as a toxicological endpoint [95].

### 3.4. Cellular and Molecular Kinetics of Short-Lifespan Models

The features of short-lifespan physiology that shape the temporal profile of PM2.5-induced pathology are elevated mass-specific metabolic rate, which accelerates reactive oxygen species generation and inflammatory pathways, and rapid cellular turnover, which accelerates both injury progression and repair. This leads to a bifurcation in outcome, determined principally by whether exposure is discrete or sustained. Where exposure is a single event, rapid turnover permits complete resolution. The murine inflammatory response to a single instillation peaked at 24 h and had returned to control values by 72 h [75]. Short-term exposure has similarly been characterized as producing transient lung injury followed by repair [97]. In this way, the tissue clears the insult before structural remodeling is established, and the model registers no chronic phenotype. Where exposure is repeated or sustained, the same rapid turnover accelerates entry into fibrotic remodeling. Time-dependent studies show progressive rather than plateauing injury: histopathological alteration, inflammatory response and fibrosis all increased with longer exposure duration, alongside cumulative DNA damage and progressive impairment of DNA repair [100]. The three-month emphysema sequence follows the same pattern [16]. Pulmonary inflammatory gene expression (including Tnf, Il1b, Il6, Ccl2 and Icam1) can persist well beyond the resolution of oxidative injury, and a subsequent re-challenge elicits an exaggerated response relative to a naive animal; this is evidence of an acquired sensitized inflammatory state rather than simple reversibility [101]. Notably, the core Jak/Jnk/NF-κB inflammatory signaling axis and systemic oxidative stress response activated by concentrated PM2.5 in Drosophila closely parallel the mammalian pattern, indicating that the fundamental molecular kinetics of particulate injury are conserved across a substantial evolutionary distance and are not an artifact specific to rodent physiology [95].

Short-lifespan animal models allow investigators to resolve the full acute injury-to-early-fibrosis trajectory, together with its underlying cellular and molecular kinetics, within an experimentally tractable window, and when calibrated to sufficient exposure intensity or observation period they can also reproduce chronic and neoplastic outcomes. What they cannot reproduce is the exposure profile itself: a low ambient concentration acting continuously across a lifespan measured in decades, under conditions of unregulated dose, uncontrolled co-exposure and biological heterogeneity. This constraint carries a direct interpretive consequence. Because the timescale is compressed to fit the animal’s lifespan, a chronic phenotype produced in a rodent is not equivalent to one produced by lifelong ambient exposure; it is in part the product of a design chosen for tractability. The evidence is also internally divided on when the endpoint should be read, since an acute inflammatory response may resolve completely after a single exposure [75,97], yet fibrosis may continue to progress for months after exposure has ceased [101]. What is recorded at sacrifice therefore depends heavily on when the animal is sacrificed, and such results should be interpreted in light of the exposure profile and observation window rather than treated as absolute. Beyond the mouse and rat that dominate the literature, other short-lifespan laboratory species remain under-used, for example, the rabbit and the hamster [104,105]. Broadening the species base would test whether current findings are general or specific to the rodent. It would not, however, resolve the anatomical objection, since efficient nasal filtration, monopodial airway branching and the scarcity of respiratory bronchioles are shared across the small laboratory species, including the mouse and rat. The other direction is to move beyond the intact animal. Ex vivo and in vitro lung models now reproduce features of the human airway that rodents cannot: three-dimensional organotypic cultures reproduce human bronchial epithelium [68]. Computational dosimetry supplies the complementary quantitative layer, with the Multiple-Path Particle Dosimetry model estimating region-specific deposited dose across species [106] and computational fluid dynamics resolving deposition differences between the human and rat nasal cavity directly [107]. None of these replaces the whole animal, since none reproduces systemic and immune integration over time, but used alongside it, they address precisely the anatomical and dosimetric mismatches that limit rodent extrapolation, and they merit a larger role in future air-pollution toxicology.

## 4. Long-Lifespan Animals: Bioaccumulation and Chronic Remodeling

### 4.1. Long-Lifespan Representative Species

Companion animals, dogs and cats, are the most studied long-lifespan animal models. They live in the same domestic environment as humans, sharing exposure to both indoor and outdoor air quality. A lifespan of more than 10 years allows cumulative particulate burden to be assessed at necropsy or through veterinary medical records [21,46,47,108,109,110]. Laboratory-based long-lifespan models are rarely used, as they require ethical permission and involve higher maintenance costs. Beagle dogs occupy a similar historical role, having anchored decades of interspecies particle-clearance and macrophage-recruitment research [36]. Non-human primates share the most anatomically similar physiology to humans and have therefore been chosen for studies on the effects of diesel and coal combustion exhaust [19]. Studies in wildlife species have also been widely conducted. Zoo or captive wildlife allow access to polluted areas associated with respiratory histopathological changes, because these animals do not relocate and have long lifespans [45]. Free-ranging wildlife also provide similar correlations through region-specific air pollution data [48]. These species are assessed by necropsy after death, which can reveal long-term tissue changes associated with the exposure setting, although attribution to PM2.5 specifically is rarely possible. These studies are observational associations. The pulmonary pathology seen in the samples may be caused by PM2.5 exposure, but because of confounding factors, a causal statement would be too strong. As Table 2 shows, the great majority of long-lifespan evidence is observational rather than experimental: companion animals are studied under the conditions to which they are actually exposed in the household environment, rather than under a researcher-administered dose. These studies also differ in the spatial resolution at which exposure is assigned, ranging from ecological (state/city-level) and regional (monitoring-station) estimates to household-level measures (indoor source inventories) and administrative data derived from population records [21,46,47,108,109,110]. The single controlled, laboratory long-lifespan comparison in the table is a 24-month chronic bioassay running cynomolgus monkeys alongside rats [19]. Necropsy- and wildlife-based studies increasingly use molecular endpoints, such as DNA methylation and histopathological grading of anthracosis, to make chronic, whole-lifetime exposure more quantitatively comparable across species and studies [45,48]. Research using long-lifespan animal models is dominated by real-world biomonitoring, including necropsy surveys, retrospective case series, and population-level health-record linkage, supplemented by a small number of historical laboratory bioassays that remain the field’s primary controlled comparator.

**Table 2:** Representative studies of PM2.5-related pulmonary and systemic effects in long-lifespan animal models, spanning real-world and laboratory settings.

Species/Model	Setting & Pollutant	Exposure Basis	Duration (≈% Median Lifespan)	Primary Phenotype/Outcome	Primary Research Focus
Dog, stray mongrel [108]	Real-world/urban ambient PM with ozone; Mexico City vs. low-pollution cities	Observational—ecological (city-level)	Lifetime (≈100%)	Innate inflammation—chronic mononuclear infiltrates Structural remodeling—bronchiolar epithelial and smooth-muscle hyperplasia; peribronchiolar fibrosis Bioaccumulation—PM-laden macrophages	Exposure-gradient comparison—canine-sentinel design contrasting between high and low pollution cities
Dog, household [47]	Real-world/traffic, household and industrial urban PM; Nigeria	Observational—ecological (state/city-level)	Lifetime; 10-yr retrospective necropsy series (2011–2020)	Bioaccumulation—high prevalence of pulmonary anthracosis with graded carbon burden	Sentinel surveillance—retrospective prevalence of pulmonary anthracosis
Dog, stray [46]	Real-world/urban ambient PM in Kathmandu Valley, Nepal	Observational—ecological (city-level)	Lifetime; clinically presenting adults	Bioaccumulation—anthracosis Clinical presentation—tachypnoea, dyspnea, cough	Sentinel surveillance—ties histological anthracosis to overt respiratory clinical signs
Dog, household [49]	Real-world/household and environmental dust; Italy	Observational—household-level exposure history	Lifetime; retrospective case–control (35 cases/160 controls)	Genotoxic/neoplastic outcome—higher anthracosis associated with primary lung cancer, OR 2.11 (95% CI 1.20–3.70) Bioaccumulation—graded anthracosis used as the exposure index	Disease-risk association—quantify carbon particle burden against cancer prevalence
Dog, cat, zoo & wildlife [45]	Real-world/habitat-specific ambient air quality; Jeollabuk-do Province, South Korea	Observational—habitat category	Lifetime; necropsy survey	Bioaccumulation—anthracosis prevalence: zoo 55%, companion 45%, wildlife 29% (habitat association, *p* < 0.05) in total 350 samples; 61 species	Exposure-gradient comparison—cross-habitat contrast isolates captivity and urban proximity
Dog & cat, companion [109]	Real-world/measured city PM2.5 levels; Bangkok, Thailand	Observational—regional monitoring records	Clinic-visit period matched to ambient PM2.5	Clinical presentation—no significant correlation between respiratory visits and PM2.5 (dog *p* = 0.057; cat *p* = 0.126)	Clinical correlation—null result attributable to statistical power and exposure contrast, not biology
Dog, working sled dogs [20]	Real-world/wildfire-smoke PM2.5; Alaska, USA	Observational—regional monitoring; repeated within-animal	Wildfire season(s); repeated measures	Oxidative stress—antioxidant capacity markers were measured, showing that exercise alone can protect antioxidant capacity	Intervention (antioxidant supplementation × exercise)—establishes sled dogs as an occupational-exposure proxy
Dog & cat, companion [21]	Real-world/indoor air pollution; Taiwan	Observational—household-level inventory	Lifetime/chronic; case–control	Clinical presentation—indoor air pollution associated with naturally acquired respiratory disease	Clinical correlation—isolates indoor from outdoor ambient PM as a distinct source
Dog & cat, companion [22]	Real-world/indoor air pollution; Taiwan	Observational—household-level inventory	Lifetime/chronic; case–control	Clinical presentation—indoor air pollution associated with cellular response and function of respiratory system	Clinical correlation—follow-up study focusing on respiratory function
Dog & cat, companion [110]	Real-world/ambient PM2.5 at population scale; UK	Observational—administrative/population records	Multi-year veterinary-visit records	Clinical presentation—higher ambient PM2.5 associated with increased veterinary visits in both species	Clinical correlation—largest sample size; care-seeking, not histopathology, as the endpoint
Grey squirrel, Sciurus carolinensis (free-ranging) [48]	Real-world/urban-to-rural pollution gradient; London, UK	Observational—site-level gradient	Lifetime; wild-caught across 4 urban and 2 rural sites	Epigenetic alteration—altered lung DNA methylation tracking the pollution gradient	Exposure-gradient comparison—Epigenetic markers and histopathological evaluation were used to compare across the sites
Cynomolgus monkey & rat (laboratory) [19]	Controlled laboratory—diesel exhaust and coal dust (surrogate particles, not ambient PM2.5)	Controlled—defined inhaled concentration	24 months (monkey ≈ 8%; rat ≈ 70%)	Structural remodeling—epithelial hyperplasia and metaplasia, greater in rats Genotoxic/neoplastic—tumors in rats, not matched in monkeys Bioaccumulation—retention site differs—rat luminal, monkey interstitial	Interspecies comparison (controlled)—the side-by-side long- vs. short-lived design; the one study that breaks the lifespan/design confound

### 4.2. Exposure Characteristics

Exposure in long-lifespan models is overwhelmingly naturalistic rather than controlled dosing, which is more easily achieved in the laboratory. Subjects are simultaneously exposed to gaseous pollutants, allergens, infectious agents, and, in the case of companion animals, indoor sources including tobacco smoke, cooking emissions, and incense [21,22]. This composite air composition is sometimes difficult to measure directly in captive or confined settings, so in some retrospective studies pollution indices are instead obtained from officially recorded data provided at the city, district, habitat-category, or monitoring-station-proximity level [20,110]. Most studies compare animals living in more- versus less-polluted geographic settings, rather than administering a defined bolus or inhaled concentration—for example, comparisons of stray dogs in Mexico City versus comparator cities [108], or decade-long retrospective necropsy series in household dogs [47]. The defining exposure variable in this literature is therefore duration rather than concentration, because these animals share the human timescale of years-to-lifetime residence in a given airshed [45,46,47]. Because concentration is rarely measured for the individual animal, differences between these findings and those from short-lifespan experiments cannot be attributed to lifespan alone. Moreover, the population is biologically heterogeneous. Outbred genetics, unknown age in many cases, variable nutritional status, intercurrent disease, and breed-associated predisposition all contribute variance that experimental designs eliminate by construction. This heterogeneity is simultaneously the principal methodological weakness of the sentinel literature and the property that makes it resemble human populations [21,22,45,47,49,109,110].

### 4.3. Long-Lifespan Model Disease Phenotypes

Pulmonary anthracosis is a visible and histologically confirmable accumulation of carbon particulate pigment [40]. It is the single most consistently reported finding across long-lifespan real-world studies, functioning as a biomarker of cumulative particulate burden that has been verified by many laboratory and human studies [40,45]. The reported prevalence of pulmonary anthracosis reaches 55% in zoo animals, 45% in companion animals, and 29% in wildlife sampled from the same region, with a statistically significant association between habitat type and the presence of anthracosis (*p* < 0.05) [45]. Similarly high prevalence has been reported in household and stray dog necropsy series from Nigeria and Nepal [46,47]. Beyond pigment deposition, chronically exposed dog lungs show a distinct low-grade, cumulative pathology: patchy chronic mononuclear infiltrates, bronchiolar epithelial and smooth-muscle hyperplasia, and peribronchiolar fibrosis [108]. Combined, these features create a pattern that resembles chronic human airway remodeling [108]. In a retrospective case–control study of 35 dogs with primary lung cancer, dogs with higher amounts of anthracosis showed an increased risk of primary lung cancer, with an odds ratio of 2.11 (95% CI 1.20–3.70; *p* < 0.01) [49]. This is an observational association from a single case–control study in which exposure was inferred from household dust history and not from measured PM2.5, so it indicates a possible relationship between retained carbon burden and lung cancer and does not show that PM2.5 caused the tumors. There is evidence relating ambient or indoor particulate concentration to clinical respiratory presentation rather than to histopathology, since histopathological changes can only be assessed after death. At the population level, ambient PM2.5 is directly associated with increased veterinary care-seeking for respiratory illness in both dogs and cats, in datasets spanning tens of thousands of animals [110]. A study conducted in Bangkok, however, found no statistically significant correlation between average PM2.5 concentration and the number of veterinary visits for either dogs (*p* = 0.057) or cats (*p* = 0.126) [109]. The explanation for this divergence is statistical power and exposure contrast rather than a biological difference between UK and Thai animals. The implication for study design is substantial: veterinary presentation is a distal, low-sensitivity endpoint influenced by owner behavior, healthcare access, seasonality, and diagnostic threshold, and detecting an ambient pollution signal through it evidently requires sample sizes in the millions [109].

Anthracosis, assessed at the gross anatomical or histopathological level, demonstrates that an animal has been exposed to and retained carbonaceous particulate, and its severity reflects cumulative dose and duration rather than recent exposure. Age is consistently associated with both the presence and the severity of anthracotic pigment, which is consistent with lifelong accumulation [45,47]. However, anthracosis cannot be read as a direct measure of PM2.5. PM2.5 is a chemically heterogeneous mixture in which carbonaceous material is only one component, so anthracotic pigment reports the retained carbon fraction rather than the mixture as a whole. The pigment is not specific to the fine fraction, since combustion-derived carbon occurs across a range of particle sizes. The relationship is therefore one of partial overlap. A heavy carbon burden implies substantial exposure to combustion-derived particulate, of which PM2.5 is typically the dominant respirable contributor, but the two are not equivalent. Demonstrating that the particles retained in a tissue are specifically PM2.5 requires direct visualization rather than routine histology. Electron microscopy and label-free optical methods resolve individual particles and, combined with elemental or spectroscopic analysis, characterize their composition in situ [111,112,113]. Only with such methods can deposition of fine particulate be claimed rather than inferred—a distinction that matters because PM2.5 translocates beyond the lung, so retained burden is not confined to the airway [13,91]. Anthracosis is nevertheless a defensible surrogate for chronic air-pollution exposure. In humans, pleural anthracosis at autopsy has been related to lifetime urban air-pollution exposure [40,42] and its quantitative analogue, carbon burden in alveolar macrophages, has been validated against measured particulate exposure and linked to lung function and disease severity [114,115]. The equivalent relationship has not been established in animals: no study has yet related semi-quantitative histopathological anthracosis grading in a companion or wildlife species to measured ambient PM2.5 at the animal’s location, because the available veterinary series assign exposure by habitat category or region rather than by monitored concentration [45,46,47].

Several chronic respiratory conditions in long-lived species are frequently discussed in association with air pollution, but a causal link to ambient PM2.5 has not yet been demonstrated for any of them. Free-ranging squirrels show altered lung DNA methylation that tracks with the urban pollution gradient, representing a distinct epigenetic phenotype not captured by the acute inflammatory endpoints that dominate short-lifespan studies [48]. Notably, head-to-head chronic inhalation bioassays show that rats develop more epithelial hyperplasia, metaplasia, and tumors than similarly exposed cynomolgus monkeys, indicating that the acute and subacute susceptibility phenotypes so prominent in short-lifespan rodents do not necessarily scale up to longer-lived, more human-comparable species [19].

### 4.4. Cellular and Molecular Kinetics of Long-Lifespan Models

The kinetic signature of long-lifespan species is fundamentally slow accumulation over a lifetime rather than rapid burst-and-resolve inflammation. Interspecies clearance studies comparing humans, baboons, beagle dogs, and several rodent strains show that the long-term insoluble particle clearance phase is far more protracted in large, long-lived species, meaning that inhaled particulate matter can reside and progressively accumulate in the lung over a much larger fraction of the animal’s life than in a mouse or rat [19]. As part of the deposition and clearance process, macrophage-mediated translocation of particles from the alveolus to the tracheobronchial lymph nodes has been directly demonstrated in dog lungs using dual-fluorescent-microsphere tracking, confirming that particle trafficking beyond the airway remains an active, ongoing cellular process over days to weeks, even as the particles themselves may persist for years [36]. Because deposition site and clearance rate differ anatomically between species, including regional differences in the centriacinar region between rodents and primates, an equivalent inhaled dose produces different regional particulate burdens and, ultimately, different histopathological trajectories across species [19].

Macrophage reprogramming under sustained particulate load has been demonstrated experimentally, with chronic carbon black ultrafine particle exposure altering macrophage metabolism and accelerating pulmonary carcinogenesis [103]. This finding is from rodents, but it offers a plausible but untested mechanistic explanation for the epidemiological association between anthracosis severity and canine lung cancer: sustained retained particulate burden maintains macrophage activation, and chronic macrophage activation is permissive for neoplastic progression [49,103]. The mechanism has been demonstrated experimentally in a short-lifespan species, whereas the corresponding outcome in a long-lifespan species is only an observational association, and the two have not been linked within the same species.

The composition of the retained burden is variable in ways that matter. Seasonal and source-related variation in the metal and polycyclic aromatic hydrocarbon content of PM2.5 produces corresponding variation in macrophage polarization and cytotoxicity, with summer-collected material in Taiwan showing the highest metal content and the greatest biotoxicity, and iron, titanium, cobalt, and antimony identified as key contributors [29]. An animal accumulating particulate matter across a decade accrues a compositionally heterogeneous burden, which no single-exposure experimental design can reproduce. The net effect is that where short-lifespan rodents resolve an acute inflammatory episode within a short period of time, long-lifespan species instead sustain a low-amplitude, slowly progressive inflammatory and fibrotic process over months to years. This is a kinetic pattern that is much harder to capture experimentally, but one that is arguably more representative of the chronic, lifetime PM2.5 exposure pattern relevant to human disease.

## 5. Comparative Analysis: Short vs. Long Lifespan

Lifespan and exposure design are confounded in the existing literature and cannot be fully separated. The lifespan frame is therefore best treated as a practical device for organizing complementary evidence rather than as a demonstrated causal determinant. Nikula et al. exposed rats and monkeys under identical controlled conditions and showed that the shorter-lived rat was more susceptible, isolating species from study design [19]. The short-lifespan rodent serves as a tractable window onto the full acute-injury-to-early-fibrosis trajectory, whereas long-lifespan companion, primate and wildlife species serve as naturalistic sentinels of cumulative particulate burden. Comparing these two models clarifies why an identical ambient PM2.5 field does not produce identical pathology across species, and why a finding that is robust in one group cannot be assumed to translate directly to the other. Three aspects account for this divergence: the anatomical and physiological variables, the time-at-risk factor, and the variance in immune response kinetics and tissue-repair efficiency. These three aspects are synthesized in Table 3 and are discussed in turn below.

### 5.1. Anatomical and Physiological Variables

Among the short-lifespan species that dominate the experimental literature, the mouse and rat, with the hamster, guinea pig and rabbit used less often, several anatomical features systematically reduce and redistribute the dose reaching the gas-exchange region. Rodents and rabbits are obligate nasal breathers, and the complex turbinate architecture of the murine and rat nasal passage acts as a pre-filter, removing a substantial proportion of inhaled particulate before it reaches the lower airway [34,116,117]. Their airways branch monopodially, with a dominant central axis giving off small lateral daughter branches [24,34]. Because impaction and sedimentation are governed by airflow velocity and directional change, this geometry shifts deposition toward different airway generations than in a dichotomously branching lung [24,107]. Most consequentially for histopathological interpretation, respiratory bronchioles are few or absent in the mouse and rat and poorly developed in the rabbit [24,116], so the centriacinar region in which human particulate pathology concentrates is largely missing from these models [118]. Physiologically, resting respiratory rate is high—approximately 272 breaths per minute in the mouse, 70–120 in the rat—and is coupled to a high mass-specific metabolic rate, so a given particulate load is processed against a faster oxidative and inflammatory background; tracheal mucociliary transport is correspondingly slow in absolute terms, at roughly 1.9 mm/min in the rat [119].

The long-lifespan sentinel species present a more human-like configuration, though not a uniform one. Dogs, cats, ferrets, pigs, sheep, horses, non-human primates and humans are oronasal breathers and lose nasal pre-filtration during oral breathing and exertion, when minute ventilation is simultaneously elevated [34,116]. Branching geometry, however, does not follow the lifespan division: non-human primates and humans branch dichotomously and relatively symmetrically, whereas the dog, cat, ferret, and pigs retain a monopodial pattern closer to that of the rodent [24,34,117]. Respiratory bronchioles are well developed in the dog, cat, ferret, non-human primate and human but sparse in the horse, pig, sheep and cattle, so possession of the principal human injury site is a species-level rather than a lifespan-level property [24,116,119]. Lobular organization adds a further aspect, since the pig, sheep and cattle have thick interlobular septa and correspondingly limited collateral ventilation, whereas the dog, cat and rodents have thin septa and extensive collateral airflow [119]. Ventilation is slower and larger in scale—15–30 breaths per minute in the dog and 12–20 in the human—with a lower mass-specific metabolic rate, and mucociliary transport is faster in absolute terms (dog 7.5–19.2 mm/min; human approximately 15.5 mm/min), although this must be read against a longer conducting airway in larger species, so clearance time rather than velocity is the meaningful quantity [119].

To summarize, three points carry most of the interpretive weight. Dose delivery differs, because rodents remove more of the inhaled burden in the nose, so an identical ambient concentration does not produce an identical delivered dose. Deposition site differs, because monopodial branching and the near-absence of respiratory bronchioles place rodent lesions in different airway generations from human lesions; lesion distribution is therefore not directly comparable even when total deposited mass is matched [107,117]. Processing rate differs, because the small species meet the aerosol at high ventilatory frequency against a fast metabolic background, whereas the long-lived species meet it in larger absolute volumes across a longer airway and a slower clearance path. The escalator is itself a target, since in one study PM2.5 reduces ciliary beat frequency and alters mucin expression independently of adsorbed chemical cargo [61]. These variables do not partition cleanly by lifespan: branching pattern groups the dog with the rodent, and respiratory bronchioles group the horse with the rodent. The anatomical axis therefore qualifies the lifespan framework rather than reinforcing it—a limitation noted in acute lung injury modeling, where failures of clinical translation have been attributed in part to anatomical and physiological differences between small mammal models and the human lung [34,88,89].

### 5.2. The Time-At-Risk Factor

Duration of exposure determines the cumulative dose over a lifetime, and it is on this point that the two groups diverge most sharply. Rodent median laboratory lifespans of roughly 730 days for the mouse and 1000 days for the rat mean that even protocols described as chronic occupy only a small fraction of the animal’s life; three to six months of continuous exposure corresponds to between one-eighth and one-quarter of a mouse’s lifespan, and the majority of representative short-lifespan models in Table 1 occupy between 0.4% and 15% of median lifespan [16,88,89]. To yield measurable pathology within a fundable timeframe, these designs therefore compress years of cumulative human exposure into weeks of elevated, often bolus, dose. The literature on long-lifespan species, by contrast, focuses on duration as the exposure variable rather than concentration; ambient concentration sustained over years of a lifetime reveals a timescale relationship to human lifetime exposure that is far more directly comparable [45]. Concentration differs by orders of magnitude between designs: the short-lifespan studies in Table 1 mostly deliver a defined, elevated dose by instillation or concentrated-particle inhalation, whereas the long-lifespan studies in Table 2 involve ambient or indoor exposure at concentrations that were rarely measured for the individual animal.

Bioaccumulation results from this long-term clearance process: inhaled particulate matter resides and progressively accumulates across a much larger fraction of the animal’s life. The histological correlate is pulmonary anthracosis. The accumulation of carbonaceous pigment functions as a cumulative marker of lifetime carbonaceous particle exposure rather than an index of recent dose, and long-lifespan experiments reveal anthracosis prevalence more prominently than the acute or chronic responses seen in short-lifespan models [40,45,46,47]. Carcinogenesis requires the accumulation of successive genetic and epigenetic alterations, and is therefore the outcome most sensitive to cumulative time at risk. Lung neoplasms developed in mice exposed to ambient-collected PM2.5 [15], and chronic carbon black ultrafine particle exposure reprogrammed macrophage metabolism and accelerated lung cancer [103]. What distinguishes the rodent from the canine case is the exposure intensity required. In dogs, a positive association between retained anthracosis and primary lung cancer emerged under ordinary ambient exposure [49]. Both species show the phenotype, but the exposure used to elicit it within a rodent lifespan is far above ambient, whereas the canine association was observed under uncharacterized environmental exposure across a natural lifespan. Critically, this susceptibility does not scale in a simple direction: in the single controlled side-by-side bioassay, rats developed more epithelial hyperplasia, metaplasia, and tumors than cynomolgus monkeys exposed concurrently for 24 months, even though the monkeys retained a similar or greater total lung burden in a more interstitial distribution [19,38]. The acute and subacute susceptibility phenotypes so prominent in rodents therefore do not necessarily predict the outcome in longer-lived, more human-comparable species.

### 5.3. Immune Response Variance

The two groups also differ in the kinetics of innate immune activation and in the efficiency of tissue repair, which together determine whether an exposure is resolved or converted into permanent remodeling. The dominant short-lifespan response is rapid, high-amplitude, and frequently self-limiting. As shown in murine studies, the inflammatory reaction to a single instillation peaks at 24 h and returns to control values by 72 h [75]. Fibrotic mechanisms have been studied using short-lifespan species; even though their rapid turnover typically resolves acute injury, chronic disease phenotypes can emerge from repeated, high-dose exposure that overwhelms the repair process. In long-lived species, the kinetic signature is slow accumulation, with a prolonged, low-amplitude inflammatory and fibrotic process sustained over months to years rather than a burst episode. Importantly, the initiating molecular machinery is conserved across this divergence: the NLRP3 inflammasome and TLR/MyD88/NF-κB pathways, onto which the heterogeneous components of PM2.5 converge, are engaged in both groups, and the core Jak/JNK/NF-κB axis is activated even in Drosophila models, indicating that the fundamental inflammatory signaling is not a rodent artifact [77,78,95]. What differs is the tempo and the anatomical compartment in which the response plays out across species.

Macrophage-mediated removal of the initial insoluble fraction is slower in humans, baboons, and dogs than in rodents [30], and the alveolar macrophage volume of the rat is substantially greater than that of the mouse or hamster, requiring a correspondingly larger engulfed burden to induce the stasis that precedes chronic retention [34]. The site of retention diverges accordingly: rats retain a greater proportion of particulate in the alveolar-duct and alveolar lumens, whereas monkeys and humans retain a greater proportion in the interstitium, and the interstitial fraction in the human lung rises with cumulative exposure from 57% in non-miners to 91% in long-service miners [19,38]. Tissue-repair efficiency helps explain this divergence as well. The rapid epithelial turnover that permits complete resolution after a single rodent insult also underlies an acquired, sensitized inflammatory state in which pro-inflammatory gene expression persists beyond the resolution of oxidative injury and a re-challenge elicits an exaggerated response [101], alongside adaptive shifts in the Th1/Th2 and Treg/Th17 balance and in immunoglobulin profiles [81,84]. The age-related difference lies mainly in defense capacity rather than initial particle reactivity, as neonatal and adult rat lungs activate antioxidant responses differently following soot exposure [120]. This susceptibility pattern may suggest that age-related differences within a species are part of a broader pattern of differential response. The long-lived lung, sustaining a lower-amplitude but continuous insult, instead trends toward slow interstitial remodeling and epigenetic changes, including altered lung DNA methylation tracking the pollution gradient in free-ranging wildlife [48], meaning this pattern better mirrors the chronic, lifetime exposure relevant to human disease.

### 5.4. Synthesis

Three aspects describe a consistent trade-off rather than a simple hierarchy of fidelity. Short-lifespan models offer anatomical tractability, genetic manipulability, and the ability to resolve fast innate and repair kinetics within an experimentally time-controlled frame, but they filter more of the inhaled dose in the nose, lack the centriacinar target region of the human lung, and cannot reproduce the exposure profile, which is a low ambient concentration acting continuously across decades, that defines human disease. Long-lifespan models supply a naturalistic version of that missing exposure profile, together with protracted clearance kinetics, interstitial particle retention in some species, human-comparable airway anatomy in some species, and chronic and neoplastic findings that arise spontaneously but are linked to exposure only through observational association, at the cost of experimental control, defined dosimetry, and biological homogeneity. These advantages should not be read as consequences of lifespan alone. The anatomical and physiological features are species-level traits that co-vary with body size and phylogeny, the time-at-risk difference is inseparable from the contrast between controlled and observational study design, and the immune and repair differences come from different species under different exposure designs. In the studies reviewed, lifespan, species, exposure concentration and duration, and study design therefore vary together, and the differences between the two groups cannot be assigned to any one of them. Neither group is a substitute for the other; each is informative about the aspects of PM2.5-induced disease that its design, exposure, and physiology allow it to express.

## 6. Methodological Challenges and Translational Value

The comparative analysis in the preceding section established that no single animal model reproduces the human condition of PM2.5 exposure, and that each group of models is informative only about the phase of disease its lifespan and physiology allow it to express. The practical consequence is a methodological dilemma that runs through the entire experimental literature.

### 6.1. Translational Mismatch

The central difficulty is one of timescale. Human PM2.5-related lung disease develops across decades of continuous, low-concentration ambient exposure, whereas the rodent that dominates the mechanistic literature has a median laboratory lifespan of only two to three years. As established in Table 1, even protocols described as chronic in rodents occupy only a small fraction of the animal’s life, and the majority of representative designs deliver their dose across between 0.4% and 15% of median lifespan [88,89]. To generate measurable pathology within this compressed window, and within the timeframe of a fundable project, researchers must raise the concentration, shorten the exposure, and sacrifice the animal at or shortly after exposure ends. This produces a systematic mismatch between the experimental exposure profile and the human exposure profile, characterized by low dose, lifelong duration, and uncontrolled co-exposure. Studies mostly terminate at or shortly after the cessation of exposure. The fibrotic process continued for eighteen months after a one-month exposure, while the oxidative stress resolved earlier [101]. As a matter of protocol, a substantial proportion of the literature therefore reports intermediate rather than final disease states.

The mismatch is not merely quantitative. Elevating the dose to compress the timeline can engage mechanisms that do not operate at ambient concentrations, such as macrophage overload, whose relevance to human carcinogenic risk at realistic exposures remains unresolved [34]. The anatomical differences noted above compound the problem, because rodents filter more of the inhaled burden in their nasal turbinates, branch their airways monopodially rather than dichotomously, and largely lack the respiratory bronchioles and centriacinar region in which human particulate pathology concentrates [24,116,118,119]. Lesion distribution is therefore not directly transferable between rodent and human even when the delivered mass is matched, and failures of clinical translation in acute lung injury modeling have been attributed in part to precisely these anatomical and physiological disparities [34,88,89]. The single controlled interspecies bioassay sharpens the caution: rats exposed concurrently with cynomolgus monkeys developed more epithelial hyperplasia, metaplasia, and tumors despite the monkeys retaining a similar or greater burden in a more interstitial distribution, indicating that the acute and subacute susceptibility phenotypes so readily produced in rodents can overpredict the response of longer-lived, more human-comparable species [19,38].

PM2.5 is not a single exposure. Composition varies by source, season, and region, and this variation produces corresponding variation in biological effect [29,55]. Studies employing commercially sourced particulate rather than locally collected ambient material may therefore not represent the exposure of the population they intend to model. This is a limitation addressed directly by the use of ambient-collected samples [15].

A limitation concerns the comparison itself. Controlled laboratory exposure and long-term environmental exposure differ in how exposure is generated, measured, and interpreted. In the laboratory, the particle source, concentration, route, and duration are chosen by the investigator, co-exposures and housing are standardized, and animals are genetically uniform and usually young at exposure, which supports causal attribution but narrows the range of conditions represented. In environmental settings, exposure is a time-varying mixture of PM2.5 with gases, allergens, tobacco smoke, and indoor sources; the concentration reaching the individual animal is usually inferred from regional monitoring or habitat category; age at exposure and exposure history are often unknown; and the endpoint is usually assessed once, at necropsy or in clinical records. The two settings therefore answer different questions. Laboratory studies show what a defined exposure can do to a defined animal, whereas environmental studies show what has accumulated in animals living in a particular place, so associations from the second cannot be read as dose–response estimates and doses cannot be transferred directly between them.

Finally, the two-year model cannot express the slow processes—progressive bioaccumulation, structural remodeling, epigenetic drift, and spontaneous carcinogenesis—that require a human-scale time-at-risk to develop. None of this diminishes the indispensability of the rodent model. Genetic tractability, defined dosimetry, controlled husbandry, and rapid, resolvable injury kinetics make short-lived models the only practical system for isolating specific molecular pathways and establishing dose–response relationships. The appropriate inference is therefore interpretive rather than dismissive: findings from short-lived models are best treated as mechanistic hypotheses about pathways and susceptibility, whose quantitative extrapolation to lifelong human ambient exposure must be validated in systems that actually experience that exposure profile.

### 6.2. One Health Perspective

Air pollution is the leading environmental risk factor worldwide and is recognized as the greatest environmental threat to human health, owing to its substantial contribution to the global burden of disease [1]. Among its constituents, PM2.5 has received particular attention in recent years because its small size and complex chemical composition together determine its capacity to cause harm. Strong evidence has established causal relationships between both acute and chronic PM2.5 exposure and all-cause mortality in humans [6], with respiratory disease, cardiovascular disease, and systemic outcomes including cancer among the principal endpoints. Respiratory effects are the most immediately evident, since the lung is the point of entry for every air-breathing organism. The consequences, however, are not confined to human health. The particles are deposited onto soil, vegetation, and surface water, where they act as a continuous, diffuse input of contaminants and nutrients to terrestrial and aquatic systems. Atmospheric deposition delivers measurable fluxes of heavy metals to agricultural soils [121], while nitrogen deposition drives acidification and nutrient enrichment in remote freshwater ecosystems [122]. Agricultural productivity is affected, depressing rice and wheat yields across large areas of China [123]; a recent multi-pollutant assessment attributed roughly 3% of the yield loss to PM2.5, partly offset by the fertilizing effect of co-deposited nitrogen [124]. Non-human animals share this exposure directly. Inhalation exposure has been associated with respiratory injury and altered physiological function across avian species [72]. Human health, animal health, and ecosystem integrity are therefore not three separate consequences of PM2.5 but three expressions of a single exposure, which is precisely the interdependence that the One Health framework was formulated to address.

One Health treats human, animal, and environmental health as interconnected, and its central observation for PM2.5 research is that companion animals occupy the human ecological niche more completely than any laboratory model can simulate [108]. Dogs and cats live in the same households and neighborhoods as their owners, breathe the same indoor and outdoor air, and share the same real-world composite exposure to ambient PM2.5 alongside tobacco smoke, cooking emissions, and incense [21,22,125]. They therefore undergo a continuous exposure that could not be administered to a rodent either ethically or practically: a low ambient concentration acting over years. Despite this shared exposure, the veterinary evidence base remains markedly underdeveloped relative to the human literature. Experimental work has been directed almost entirely toward short-lived rodent models selected to represent disease mechanisms, whereas naturally exposed veterinary species have seldom been studied as subjects in their own right, and the reports that do exist are comparatively few, are often confined to single sites or small case series, and rarely incorporate quantified individual exposure data. This scarcity matters because it limits the veterinary profession’s capacity to recognize and manage pollution-related disease in its own patients, and it leaves a substantial body of naturally occurring, chronically exposed cases unexploited as evidence relevant to human health.

Several features make the companion animal a uniquely suitable sentinel. Its exposure is naturalistic rather than a researcher-administered bolus, so the delivered dose, co-exposures, and biological heterogeneity resemble those of the human population. Its lifespan is long enough to permit the bioaccumulation, chronic remodeling, and spontaneous neoplasia that short-lived rodents rarely reach, yet short enough to compress disease latency into an observable window [126]. Because disease arises spontaneously rather than being experimentally induced, the sentinel captures authentic gene-environment interactions, and companion animals carry fewer of the occupational, smoking, and alcohol confounders that complicate human epidemiology, sharpening the exposure-response relationship [126]. Dogs breathe nearer the road surface than their owners, which may expose them to higher concentrations of resuspended road dust and traffic emissions, although this has not been quantified in this context. An animal accumulating a compositionally heterogeneous particulate burden across a decade thus provides an integrative record of lifetime exposure of a kind no single-exposure experiment can reproduce.

The sentinel approach carries its own methodological challenges, however, and honest appraisal of them is essential to its translational value. Individual exposure is rarely measured directly; it is usually inferred from city-, district-, or monitoring-station-level data, so exposure misclassification is a persistent risk [20,110]. Definitive histopathological endpoints such as anthracosis grading can generally be assessed only at necropsy, restricting antemortem study to distal, low-sensitivity outcomes; the divergence between a large-sample study detecting an ambient-PM2.5 signal in veterinary utilization and a smaller-sample study finding no significant correlation is best explained by statistical power and exposure contrast rather than a biological difference, and implies that population-level sentinel signals may require very large cohorts to resolve [109,110]. The outbred heterogeneity that makes companion animals resemble human populations simultaneously introduces variance that controlled experiments eliminate. Zoo collections add further strengths to this approach: known age, documented residence duration at a fixed location, and systematic necropsy. Their inclusion also extends the taxonomic range enormously, permitting the lifespan hypothesis to be examined across a range of longevities [45].

These considerations point toward an integrated rather than a competitive use of models. The short-lived rodent supplies mechanistic causality under controlled conditions but cannot reproduce the human exposure profile; the long-lived companion animal supplies that exposure profile, human-comparable anatomy, and spontaneous chronic disease but sacrifices experimental control. Within a One Health framework, their weaknesses are complementary: mechanisms defined in rodents can be interrogated for their real-world footprint in sentinel species, and associations observed in sentinels can be referred back to rodents for mechanistic dissection. The mechanistic model, naturalistic sentinel, and human observation combine to form the most defensible route to translating PM2.5 toxicology into human health protection, and this is the central methodological argument that follows from the lifespan-based comparison.

## 7. Conclusions

This review set out to contrast the phenotypic, cellular, and temporal manifestations of PM2.5-induced lung disease between short- and long-lifespan animals, and the evidence assembled points to a single organizing conclusion. The mechanisms of PM2.5 toxicity are broadly conserved, but the pathology they produce is partitioned by lifespan into two distinct phenotypic poles. The heterogeneous components of PM2.5 converge on a shared set of innate and adaptive inflammatory pathways. What differs between species is the phenotype into which this activation resolves. In short-lifespan models, the dominant expression is an acute, inflammatory phenotype that, following a discrete exposure, frequently resolves within days [75,97]. In long-lifespan species, the dominant expression is instead chronic bioaccumulation, registered as pulmonary anthracosis, airway remodeling resembling the human pattern, and spontaneously arising lung cancer [45,49,108]. These patterns are not mutually exclusive, because short-lived models can develop chronic phenotypes given sufficient dose or observation time [15,18,88]. In the one controlled comparison, the short-lived rat was more susceptible than the long-lived monkey [19]. The evidence therefore does not support attributing the phenotypic difference to lifespan itself, which remains confounded with study design and with exposure intensity and duration.

The two model groups are best viewed as complementary rather than competing sources of evidence. Short-lifespan models offer what controlled systems do best: a known dose and composition over a known interval, which supports causal attribution, genetic manipulation to test pathway necessity, and serial sampling to establish the order of events. Their main contribution is mechanistic resolution. Long-lifespan animals, in the largely observational studies available, offer a naturalistic composite exposure at ambient concentration over years, in outbred animals carrying comorbidities and co-exposures, with airway dimensions, ventilation and clearance kinetics closer to humans. Their main contribution is exposure realism, although causal attribution is limited. Because lifespan remains confounded with study design and with exposure intensity and duration, these differences should not yet be attributed to lifespan alone. The value of each is greatest when read against the other: a pathway defined in rodents gains public-health meaning when its footprint is found in naturally exposed populations, and an association in a sentinel species gains causal interpretation when its mechanism can be tested experimentally. The practical value of a lifespan-based framework is therefore organizational: it allows researchers to reframe apparently conflicting findings as complementary windows onto a single disease process rather than as contradictions to be reconciled.

**Figure 2:**
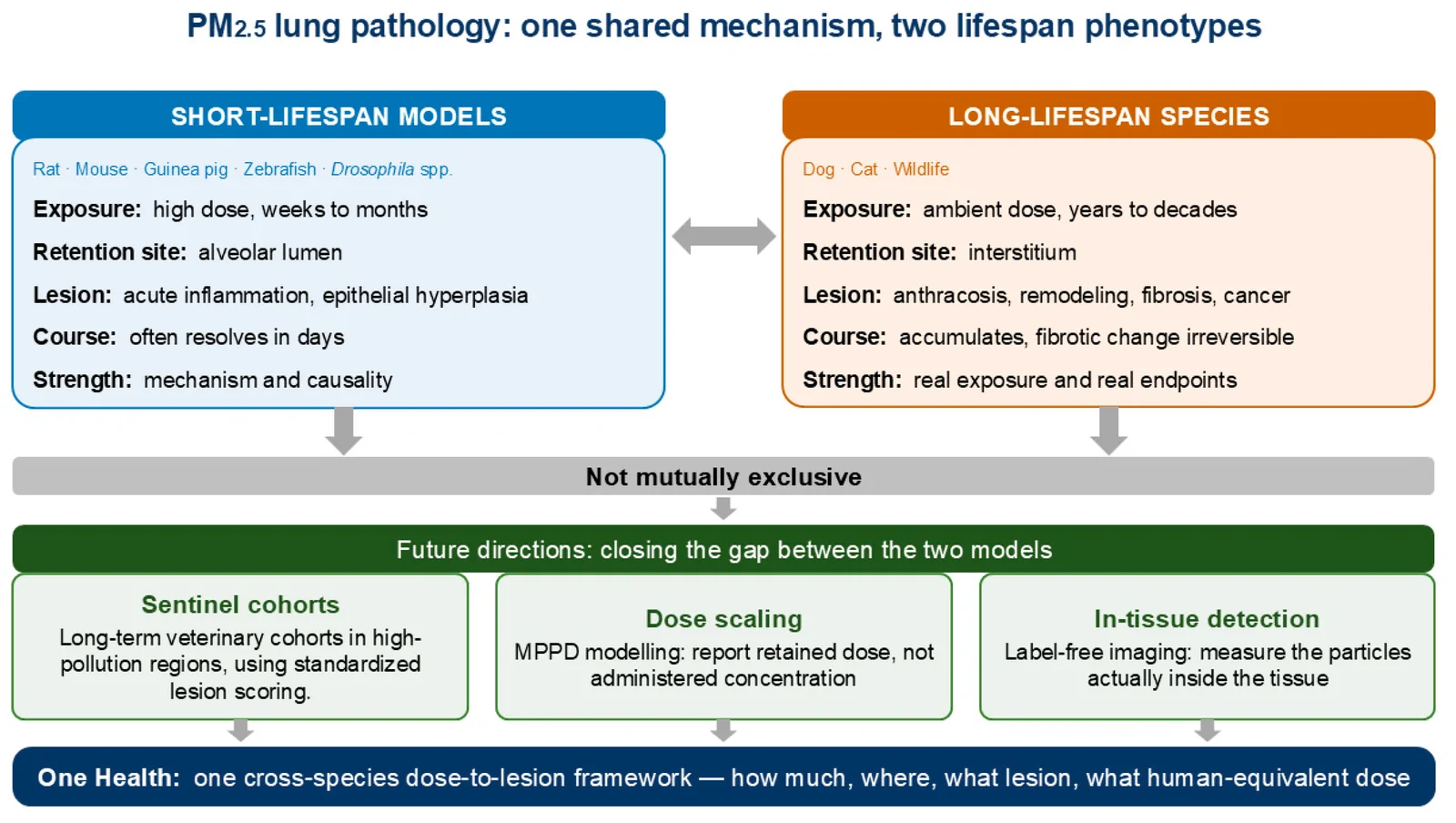
Summary of the lifespan-based framework and the research priorities that follow from it. Short- and long-lifespan models are associated with different phenotypes of a shared mechanism. Left, short-lifespan models (rat, mouse, zebrafish, and Drosophila, together with the guinea pig, which has an intermediate lifespan and is grouped here because it was studied under the same controlled-exposure design) are characterized by controlled, usually high-dose exposure over weeks to months, acute inflammatory and early fibrotic lesions that often resolve, and strength in mechanism and causal inference (Section 3). Right, long-lifespan species (dog, cat, and wildlife) are characterized by observational studies of ambient or household exposure over years to decades, with anthracosis, remodeling, and fibrosis as the main findings and lung cancer reported only as an observational association in dogs (Section 4); these entries should be read with the qualifications given in Section 4.3 and Section 5. The double-headed arrow and the label “Not mutually exclusive” indicate that rodents can develop chronic and neoplastic lesions at sufficient dose or duration and that lifespan is confounded with exposure design (Section 5). Bottom, three research priorities (Section 7.1, Section 7.2 and Section 7.3), namely long-term veterinary sentinel cohorts, dose scaling, and in-tissue detection, converge on a cross-species dose-to-lesion framework within a One Health perspective.

Three developments would close this distance, and each addresses a specific limitation exposed by the lifespan comparison: extending the sentinel evidence base where the long-lifespan phenotype is most fully expressed, establishing a dosimetric currency in which short- and long-lifespan exposures can be equated, and measuring retained particle burden directly in tissue so that dose and lesion can be linked within the same specimen. The lifespan-based framework and these three priorities are summarized in Figure 2.

### 7.1. Long-Term Veterinary Epidemiology in High-Exposure Regions

The sentinel value of long-lived companion animals is currently constrained by where the studies have been done. The necropsy studies from Kathmandu and southwest Nigeria, and the clinic-linked data from Bangkok, demonstrate both the feasibility and the interpretive difficulty of relating air pollution to animal health [46,47,109]. Southeast and East Asia, where ambient concentrations are among the highest globally and where seasonal biomass burning produces steep temporal exposure gradients suitable for within-population comparison, remain substantially underrepresented. What is needed next are prospective, long-term veterinary cohorts in high-pollution zones that link individually or regionally measured ambient PM2.5 to standardized histopathological endpoints at necropsy, rather than to distal proxies such as clinic attendance, whose low sensitivity demands very large samples [109,110]. Such studies would directly exploit the One Health premise that companion animals are co-exposed, shorter-latency sentinels of the human population [126]. Their translational value also depends on methodological standardization—in particular, consistent, semi-quantitative grading of anthracosis and associated lesions—so that burden can be compared across regions, species, and studies [45,127].

### 7.2. Quantitative Dose-Scaling Models

The identified translational mismatch is ultimately a dosimetric problem, and it is most defensibly addressed with computational dose modeling rather than with the administered concentration that dominates current reporting. Mechanistic deposition models such as the Multiple-Path Particle Dosimetry (MPPD) model already compute region-specific deposited and retained particle dose in the airways of both rats and humans from species-specific airway geometry, ventilation, and particle characteristics, and thereby provide a principled basis for converting a rodent exposure into its human-equivalent delivered dose [106]. Reporting modeled deposited dose per unit alveolar surface area, alongside ambient concentration, would materially improve comparability at negligible cost. These future models should normalize cross-species findings to retained rather than administered dose, incorporate the interspecies differences in clearance kinetics and interstitial retention previously described [19,30], and add an explicit time-at-risk dimension that scales the compressed weeks-to-months of a rodent protocol against the decades of human ambient exposure. Coupling deposition models with physiologically based clearance-and-retention compartments, and anchoring their outputs to measured tissue burden, would allow laboratory dose–response data to be projected onto long-lived clinical realities with quantified uncertainty rather than by qualitative analogy. It would also convert lifespan from a confounder that separates the two literatures into a parameter that connects them.

### 7.3. Direct In-Tissue Detection of PM2.5 and Integration into a Dose-to-Lesion Framework

Rather than inferring dose from ambient concentration, some recent studies have focused on directly measuring the particle burden present in a cell or tissue and correlating it with exposure and pathological change. Several emerging label-free imaging technologies now make this feasible. Fluorescence lifetime imaging microscopy (FLIM) exploits the very short fluorescence lifetime of ultrafine particulates relative to tissue autofluorescence to detect and quantify PM directly in lung and heart tissue, and has been used to relate particle load to concurrent markers of inflammation, amyloid deposition, and fibrosis in the same sections [128]. White-light generation under femtosecond pulsed illumination provides a complementary, highly specific readout for carbonaceous (black-carbon) particles, enabling label-free detection and quantification of combustion particles in human tissue and their use as an internal-dose biomarker [111]. These optical methods can be paired with electron microscopy for nanoscale localization of particles within cells and, where elemental or molecular speciation is required, with energy-dispersive X-ray and Raman spectroscopy to fingerprint the metal and carbon composition of the retained burden in situ [112]. In situ detection is not limited to carbonaceous particles: Fe_3_O_4_ magnetic nanoparticles have been detected directly in the lung tissue of subway commuters, and inhalation of these particles injured the lungs of mice [59]. This supports replacing concentration-based inference with in situ quantification of the retained burden, and shows that the particles retained in the lung include metal-bearing components as well as carbonaceous ones.

Correlating each PM compartment with cellular response is a widely used approach in air pollution toxicity research. The carbonaceous compartment can be detected in biological samples, such as tissue, using optical techniques combined with spectrometry to further characterize its composition [4]. Metal composition can similarly be identified in PM2.5 samples using techniques such as ICP-MS or EDAX to assess its impact on cellular or systemic response [29,57,58]. Compartment analysis of PM2.5 in the extracellular matrix (ECM) and in ambient PM2.5 allows researchers to show that the lung immune system’s primary function is detoxification by removing components identifiable by immune cells; the remaining PM is then relocated and deposited into the ECM [113]. Methods that directly link particle burden to histopathological changes will therefore be essential to revealing how PM2.5 effects progress from the cellular to the clinical level.

The most valuable development would be to integrate these capabilities into a single, cross-species dose-to-lesion framework with three linked layers: direct in-tissue quantification of retained particle burden by label-free imaging; standardized, ideally digitally assisted, quantitative histopathology of the associated lesions using consistent scoring of anthracosis, inflammation, fibrosis, and neoplasia [127]; and computational dosimetry that ties the observed tissue burden back to an inhaled exposure and forward to a human-equivalent dose [106]. Such a framework would allow a researcher to state, for a given specimen, how much particulate is present, where it is deposited, what pathology accompanies it, and what human exposure that burden corresponds to. In doing so, it would resolve much of the current uncertainty by replacing the administered-concentration surrogate that underlies it. Applied across the short- and long-lifespan models compared in this review, it offers a concrete route to reconciling their divergent phenotypes within a common quantitative currency, and thereby to translating PM2.5 toxicology into protection of human respiratory health.

Taken together, these three directions would convert the lifespan-based comparison from a descriptive observation into a working methodology: sentinel cohorts sited where the chronic phenotype is expressed, a dosimetric scale that carries both dose and time, and an in-tissue measurement common to every species. The conserved mechanisms of PM2.5 toxicity are unlikely to be revised substantially by further work; what remains unresolved is how those mechanisms are expressed across the range of exposures and lifespans that real populations experience. Within a One Health framework, in which humans, companion animals, and the shared environment are understood as a single exposed system, resolving that question in animals is not an approximation of human research but a constituent part of it.

## Figures and Tables

**Table 3:** Comparative synthesis of short- versus long-lifespan animal models of PM2.5-induced pulmonary pathology, across anatomical and physiological variables, the time-at-risk factor, and immune response variance. NHP, Non-human primate.

Variable	Short-Lifespan (Mouse, Rat)	Long-Lifespan (Dog, Cat, Ferret, NHP, Livestock, Wildlife, Human)	Comparability
Anatomical and physiological			
Lifespan (median)	Short (mouse ~2 yr; rat ~3 yr)	Long (dog >10 yr; NHP and wildlife years to decades; human ~70+ yr)	Not directly comparable. Weeks in a rodent are not decades in a human, so exposure should be expressed as a percentage of lifespan. Short-lifespan models are also studied in controlled experiments with a defined exposure time, whereas long-lifespan species are studied observationally, so lifespan is confounded with study design and exposure time.
Breathing rate (resting, breaths/min)	Fast (mouse ~272; rat 70–120)	Slow (dog 15–30; human 12–20)	Rodents breathe and metabolize faster than species with slower breathing rates, so oxidative injury per gram of lung may build sooner and effects may appear earlier. This reflects body size and metabolic rate, not lifespan.
Ventilation (minute volume, absolute)	Small (low air volume per unit time)	Large (high air volume per unit time)	The same air concentration does not mean the same delivered dose. Ventilation differs with body size, not with lifespan.
Breathing route	Nasal only (turbinates pre-filter)	Nasal and oral in dog, cat, ferret, NHP, and human (filtration lost when panting or exercising)	Rodents remove more particles in the nose before the lower airway, so their lower-airway dose is lower than the ambient concentration implies. Oronasal breathers lose nasal pre-filtration during oral breathing and exertion, so more particles can reach the lower airway. This is a species trait.
Airway branching	Monopodial	Mixed: dichotomous (human, NHP); monopodial (dog, cat, ferret, and pigs)	Does not follow lifespan. Deposition sites differ, so lesion location cannot be mapped one to one.
Respiratory bronchioles (centriacinar region)	Absent or few (mouse, rat); poorly developed (rabbit)	Well developed (dog, cat, ferret, NHP, human); sparse (horse, pig, sheep, cattle)	Does not follow lifespan. The main human injury site is missing in rodents.
Lobular organization (collateral ventilation)	Thin septa (extensive collateral airflow)	Mixed: thin septa (human, dog, cat); thick septa with limited collateral flow (pig, sheep, cattle)	Lobular organization is a species-specific characteristic. It affects how deposited particles redistribute within a lobule.
Mucociliary clearance (transport velocity)	Slow (rat ~1.9 mm/min)	Fast (dog 7.5–19.2; human ~15.5 mm/min)	Airway length differs between species, so clearance time, not speed, is the comparable quantity. This is a species trait.
Time-at-risk			
Exposure duration (% of lifespan)	Short (~0.4–15%; high or bolus dose)	Lifelong in dogs, cats, and zoo or wildlife animals, not measured per animal (continuous, low dose)	Rodent studies compress years into weeks. A short high dose is not equivalent to a lifetime low dose.
Exposure concentration and route	High or bolus dose by instillation or concentrated-particle inhalation	Ambient or indoor air; concentrations rarely measured for the individual animal	Concentration, and not lifespan alone, may explain differences in lesion severity. The same ambient concentration does not imply the same delivered dose.
Study design	Controlled (laboratory inhalation or instillation)	Observational (biomonitoring, necropsy survey)	Trade-off: rodents give dose control, long-lived species give real-world exposure.
Insoluble particle clearance	Fast (rapid clearance phase)	Slow (prolonged retention, progressive accumulation)	Long-lived lungs accumulate across a larger fraction of life. Retained dose, not inhaled dose, drives chronic disease phenotype.
Bioaccumulation marker (anthracosis)	Rare at ambient dose	Anthracosis reported in dogs, cats, and zoo and wildlife animals; observational, with exposure assigned by habitat or region	Anthracosis reflects cumulative, not recent, exposure. It reflects retained carbon and not PM2.5 specifically, and is usable as a surrogate index of cumulative air-pollution exposure.
Neoplasia (dose required)	High exposure (above-ambient dose needed within lifespan)	Ambient exposure (dog: anthracosis associated with primary lung cancer in a single study of 35 cases; observational association only)	Rodents develop tumors at high dose; the canine evidence is a single observational association; in the only controlled comparison, rats developed more tumors than monkeys.
Structural remodeling	Burst-and-resolve (fibrosis at intense or repeated dose)	Slowly progressive (dog: bronchiolar hyperplasia and peribronchiolar fibrosis, observational)	Only long-lived species reproduce the chronic human pattern. Rodent recovery can mask long-term risk.
Immune response and repair			
Inflammation kinetics	Fast burst (peaks 24 h, resolves by 72 h)	Slow and sustained (low amplitude, months to years; dog: chronic mononuclear infiltrates)	A rodent timepoint captures the burst; a long-lifespan sentinel captures the accumulation.
Macrophage clearance rate	Fast	Slow (human, baboon, dog)	Long-lived species hold particles longer, so the same deposited dose becomes a larger retained dose.
Dominant retention compartment	Luminal in rat (alveolar ducts and alveoli)	Interstitial (human: 57% in non-miners rising to 91% in long-service miners)	Lesion location differs even when total burden matches. Interstitial retention is what anthracosis and fibrosis follow.
Tissue repair capacity	High turnover (full resolution, but sensitized to re-challenge)	Slow repair (progressive remodeling in dogs; DNA methylation change in free-ranging grey squirrels)	Rodents recover and can mask chronic risk; long-lived species convert repeated injury into fixed disease.

## Data Availability

No new data were created or analyzed in this study. Data sharing is not applicable to this article.

## Abbreviations

The following abbreviations are used in this manuscript:

AT1/AT2	Alveolar type 1/type 2 cell
BALF	Bronchoalveolar lavage fluid
BC	Black carbon
BrC	Brown carbon
COPD	Chronic obstructive pulmonary disease
EC	Elemental carbon
GSH/GSSG	Reduced/oxidized glutathione
IL	Interleukin
LPS	Lipopolysaccharide
MAPK	Mitogen-activated protein kinase
NF-κB	Nuclear factor kappa B
NHP	Non-human primate
NLRP3	NLR family pyrin domain containing 3
OC	Organic carbon
OVA	Ovalbumin
PAHs	Polycyclic aromatic hydrocarbons
PM2.5	Particulate matter ≤ 2.5 µm in aerodynamic diameter
PRR	Pattern recognition receptor
ROS	Reactive oxygen species
TGF-β	Transforming growth factor beta
TLR	Toll-like receptor
TNF-α	Tumor necrosis factor alpha
